# Antigen-specific activation of gut immune cells drives autoimmune neuroinflammation

**DOI:** 10.1080/19490976.2025.2601430

**Published:** 2025-12-24

**Authors:** Lena K. Siewert, Kristina Berve, Elisabeth Pössnecker, Julia Dyckow, Amel Zulji, Ryan Baumann, Aida Munoz-Blazquez, Gurumoorthy Krishnamoorthy, David Schreiner, Sharon Sagan, Charlotte Nelson, Joseph J. Sabatino, Kazuki Nagashima, Médéric Diard, Andrew J. Macpherson, Stephanie C. Ganal-Vonarburg, Michael A. Fischbach, Scott S. Zamvil, Lucas Schirmer, Sergio E. Baranzini, Anne-Katrin Pröbstel

**Affiliations:** a Departments of Neurology, Biomedicine and Clinical Research, University Hospital and University of Basel, Basel, Switzerland; b Research Center for Clinical Neuroimmunologyand Neuroscience Basel (RC2NB), University Hospital and University of Basel, Basel, Switzerland; c European Society for Clinical Microbiology and Infectious Diseases (ESCMID), Basel, Switzerland; d Department of Neurology, Medical Faculty Mannheim, Heidelberg University, Mannheim, Germany; e Interdisciplinary Center for Neurosciences, Heidelberg University, Heidelberg, Germany; f Department of Neurology & Weill Institute for Neurosciences, University of California, San Francisco, US; g Center of Neurology, Department of Neuroimmunology, University Hospital and University Bonn, Bonn, Germany; h German Center for Neurodegenerative Diseases (DZNE), Bonn, Germany; i Program in Immunology, University of California, San Francisco, US; j Department of Bioengineering, Stanford University, Stanford, CA, US; k Department of Microbiology and Immunology, Stanford University School of Medicine, Stanford University, Stanford, CA, US; l ChEM-H Institute, Stanford University, Stanford, CA, US; m Chan Zuckerberg Biohub, San Francisco, CA, US; n Biozentrum, University of Basel, Basel, Switzerland; o Department of Visceral Surgery and Medicine, Inselspital, Bern University Hospital, University of Bern, Bern, Switzerland; p Department for BioMedical Research, University of Bern, Bern, Switzerland; q Mannheim Center for Translational Neuroscience, Medical Faculty Mannheim, Heidelberg University, Mannheim, Germany; r Mannheim Institute for Innate Immunoscience, Medical Faculty Mannheim, Heidelberg University, Mannheim, Germany; s Institute for Human Genetics, University of California, San Francisco, US; t Graduate Program in Bioinformatics, University of California, San Francisco, US

**Keywords:** Microbiome, multiple sclerosis, neuroinflammation, mucosal immunology

## Abstract

Microbiome-based therapies are promising new treatment avenues. While global alterations in microbiota composition have been shown in multiple sclerosis, whether and how gut microbiota influence autoimmune responses in an antigen-specific manner is unclear. Here, we genetically engineered gut bacteria to express a brain antigen and dissect their pathogenic potential in a murine model of autoimmune neuroinflammation. Colonization with bacteria expressing myelin - but not ovalbumin-peptide exacerbates an encephalitogenic immune response in the gut by activating antigen-specific T cells as well as B cells leading to accelerated neuroinflammatory disease. These results demonstrate how antigen-specific microbial modulation can influence autoimmunity, providing insight for development of therapeutic strategies targeting specific bacterial taxa for treatment of MS and other autoimmune diseases.

## Introduction

With the increasing interest in harnessing gut microbiota as therapeutics, tremendous advancements in engineering bacteria for various clinical and preclinical applications have been made. These include activation of the immune system in the context of cancer or tolerance induction in autoimmune diseases.^1^^,^^2^ In addition, different strategies to restore microbiome homeostasis are being developed.^3-5^

In that regard, alterations in gut microbiota composition and its influence on immune cell (dys-) regulation have been demonstrated in the pathogenesis of several immune-mediated disorders,^6^ including multiple sclerosis (MS).^7-11^ As the most common demyelinating autoimmune disease of the central nervous system (CNS), MS is a prototypic neuroinflammatory condition for which commensal bacteria may influence or even drive the autoimmune response targeting myelin.^7-9^

While some reports have suggested an antigen-independent role of gut commensals in experimental models of neuroinflammation,^11^^,^^12^ potential molecular mimicry between gut commensals and distant tissues has recently been implicated in various (auto-) immune diseases, including MS.^13-17^ Previous studies have shown that the administration of microorganisms engineered to express myelin antigens, which do not induce strong inflammatory immune responses, attenuated experimental autoimmune encephalomyelitis (EAE).^18^^,^^19^ However, different bacterial strains within the gut microbiota possess varying capacities to stimulate immune responses, some inducing regulatory immune responses while others induce pro-inflammatory responses, which can influence disease outcomes.^20-22^

To this end, we engineered an Δ*aroA*-attenuated strain of *Salmonella enterica* to express the myelin oligodendrocyte glycoprotein (MOG) peptide using a bacterial outer membrane expression system.^23^^,^^24^ This auxotrophic strain, although not a classical commensal organism, has been used to engineer heterologous antigens and is known to elicit complex mucosal immune responses, providing a genetically tractable platform to study antigen-specific immune activation in an inflammatory gut environment. Utilizing a murine MOG-transgenic T cell receptor (TCR) model of MS, we explored if colonization with MOG peptide-expressing bacteria can lead to an antigen-specific commensal-directed immune response in the intestine and subsequently induce autoimmune neuroinflammation.

## Methods

### Bacterial expression constructs

To generate a vector expressing a concatemer of CD4-T cell epitopes from OVA (OVA_323-339_) or murine MOG (MOG_35-55_) on the surface of attenuated *Salmonella enterica* Δ*aroA* SL7207 ^25-27^ and *E. coli* MP1,^28^ we utilized the previously published plasmid pZS1205-4 containing four OVA_323-339_ concatemers fused to the MisL autotransporter and the plasmid backbone pZS1205 to design the MOG vector *de novo.*^24^ Oligonucleotides corresponding to the plus and minus strands of the MOG_35-55_ coding sequence were annealed *in vitro*, incorporating *NheI* and *XbaI* restriction sites at the 5′ and 3′ ends, respectively. The annealed mini-gene was digested with *NheI* and *XbaI* (New England Biolabs) and ligated into *NheI/XbaI*-linearized pZS1205 using T4 DNA ligase (New England Biolabs). Individual clones were confirmed by Sanger sequencing. Bacteria carrying the generated plasmids were grown in ampicillin-containing selective medium.

### Bacterial strain generation and handling

Electrocompetent *Salmonella* or *E. coli* were transformed with the respective plasmids, and clones were confirmed by Sanger sequencing. To verify successful expression and proper extracellular localization of the OVA- or MOG-epitope, bacteria expressing the fusion proteins were analyzed by western blot and bacterial flow cytometry. *Salmonella* or *E. coli* strains were cultured overnight at 37 °C in LB medium containing ampicillin (100 μg/ml). For co-culture assays, bacteria were heat-inactivated by adjusting cultures to OD_1_ in PBS, followed by incubation at 70 °C for 1 h.

### Western blot

Bacteria were grown overnight, washed in PBS, and resuspended at OD 0.1 cells per 100 µl in 1 x Laemmli buffer (BioRad). Samples were boiled at 95 °C for 10 min, then loaded onto SDS-PAGE gels and transferred onto membranes. Blots were stained with anti-OVA_323-339_ (Innovagen) and anti-MOG_35-55_ (NYRMOG, Santa Cruz) primary antibodies, followed by anti-rat/mouse-HRP secondary antibody (BioLegend). Detection was performed via chemiluminescence.

### Bacterial flow cytometry

Bacterial flow cytometry was performed as previously described.^29^ In brief, 1 million live bacteria were stained in staining buffer (PBS containing 1% (w/v) bovine serum albumin (BSA, Sigma)) with either mouse serum (1:50) or anti-OVA_323-339_ (Innovagen) and anti-MOG_35-55_ (NYRMOG, Santa Cruz) IgG, and incubated for 1 h on ice. After washing, the bacteria were stained with buffer containing the respective secondary antibodies for 30 min on ice. For staining with murine serum, anti-mouse IgG-BV421 (BioLegend), anti-mouse IgM-APC (Miltenyi), and anti-mouse IgA-PE (Southern Biotech) were used. For MOG staining anti-mouse IgG-AF647 (Biolegend) and for OVA staining anti-rabbit-PE (BioLegend) were used. Samples were then washed three times, fixed with 4% PFA and analyzed by flow cytometry on a Cytoflex (Beckman Colter). Data analysis was performed using FlowJo (BD) software.

### Animal housing

For specific pathogen-free (SPF) mice, C57BL/6 CD45.1 (*B6.SJL-Ptprca Pepcb/BoyJ*),^30^ OT-II (*B6.Cg-Tg(TcraTcrb)425Cbn/J*),^31^ and TCR^MOG^ mice (*2D2; C57BL/6-Tg(Tcra2D2,Tcrb2D2)1Kuch/J*)^32^ were purchased from Jackson Laboratory. Mice were maintained and bred at the UCSF SPF animal facility and at the TCMF, University of Basel, Basel, Switzerland. Germ-free (GF) TCR^MOG^ mice were rederived from SPF strains at the Clean Mouse Facility in Bern, Switzerland, and bred and maintained in sterile isolators, assayed regularly for GF status.

All animal protocols were approved by the Institutional Animal Care and Use Committee, the Laboratory Animal Resource Center, and the Cantonal Veterinary Offices of Bern and Basel, Switzerland, under license number 3143_34428. Mice were housed in closed caging systems, provided with standard irradiated chow diet and water ad libitum, and kept under a 12 h light cycle. For colonization of transgenic mice, both sexes were equally distributed among treatment groups. Experimental autoimmune encephalomyelitis (EAE) immunizations in WT mice were exclusively performed using female mice. In accordance with the 3 R principles, to reduce, replace, and refine animal experimentation, experiments were designed to use as few mice as possible while ensuring interpretable results. Transgenic TCR^MOG^ mice were used with a cohort size of 3-5 mice per experimental group, with 3-4 repetitions. For classical WT EAE immunizations, which are known to have more variation within groups, 8-10 mice per experimental group were used.

### Mouse colonization and EAE induction

For SPF mice, engraftment of antigen-expressing *Salmonella* was performed as previously described.^28^ Briefly, mice (6−8 weeks old) were given streptomycin and glucose in drinking water (5 mg/ml) for 72 h, followed by a 24 h washout phase with regular water. Mice were then orally inoculated with 10^9^ CFU of living bacteria in PBS from an overnight culture by gavage. Pertussis toxin (300 ng, Merck) was administered i.p. immediately after gavage and 48 h later. For GF mice, 4-5-week-old TCR^MOG^ mice were gavaged three weeks before the first pertussis toxin injection, repeated after 48 h. For WT EAE immunization, 100 µg MOG_35-55_ (Anaspec) in 100 µl CFA emulsion was injected s.c. (2 × 50 µl on both sides) on day 21 post-colonization, with pertussis toxin given i.p. and repeated after 48 h. Colonization was monitored by plating stool samples on LB plates with ampicillin (100 µg/ml). Mice were scored daily on a 5-point scale: 0, no deficit; 1, limp tail only; 2, limp tail and hind limb weakness; 3, complete hind limb paralysis; 4, complete hind limb paralysis and partial/complete forelimb paralysis; 5, death.

For histopathological analysis of disease, organs from SPF and GF mice were harvested 14 days post-first pertussis toxin injection (peak disease). For scRNA-seq and immunophenotyping by flow cytometry, organs from Sal-MOG vs. Sal-OVA mono-colonized GF mice were harvested 3 weeks post-colonization (baseline phenotyping). To compare Sal-MOG with *E. coli* colonized mice, immunophenotyping was performed on day 14 post-pertussis toxin injection (peak disease). For bacterial colonization analysis, organs were harvested at specified time points. Mesenteric lymph nodes, spleen, Peyer’s patches, and feces were homogenized using 70 μm cell strainers (BD Biosciences), plated on LB plates with ampicillin, and CFUs were counted after incubation at 37 °C.

### Behavioral assessment

Rotarod (Ugo Basile) testing was performed on a rotating rod that accelerated from 0 to 40 rotations per minute (rpm) during a 5-minute period. The latency to fall (in seconds) was recorded for each mouse to assess motor deficits and endurance. Mice were tested three times per day for three consecutive days.

### *In vitro* antigen stimulation assay

For dendritic cell (DC) expansion, murine B16 melanoma cells expressing Fms-like tyrosine kinase 3 ligand (B16-Flt3L) were thawed and cultured in DMEM (Gibco) with 10% FCS and 1% PenStrep at 37 °C. A total of 5 × 10^6^ live cells were suspended in 1 ml PBS and injected i.p. into C57BL/6 CD45.1 mice.^33^^,^^34^ After 12 days, spleens were harvested and manually homogenized. After RBC lysis, CD11c-positive DCs were extracted via positive selection using a CD11c microbead kit (Miltenyi). CD11c-positive cells were resuspended at 1 × 10^7^ cells/ml in DMEM.

For splenic T cell extraction, spleens from 2D2 or OT-II mice were harvested, and after RBC lysis, splenic T cells were extracted via negative selection using a T cell isolation kit (Miltenyi). T cells were resuspended at 1 × 10^6^ cells/ml in 5% FBS DMEM.

As previously described, DCs (50 µl at 1 × 10^7^ cells/ml) were cultured with 10 µl heat-inactivated bacteria in PBS (OD_1_) or 1 µg of peptides for 1-2 h at 37 °C.^35^ Splenic T cells (50 µl at 1 × 10^6^ cells/ml) were added and incubated with DCs for 24 h. To assess cytokine production, brefeldin A (5 ug/ml, BD Biosciences) was added during the last 4 h of incubation. Cells were stained with viability dye (Zombie UV, BioLegend, or eBioscience™ Fixable Viability Dye eFluor™ 506, Invitrogen), Fc blocker (Miltenyi), and antibodies for surface markers: BV650-anti-CD45.1, PerCP/Cy5.5-anti-CD4, FITC-anti-TCR Vβ11, and, depending on the panel, APC/Cy7-anti-CD25, APC-anti-CD44, PE-anti-CD62L, BV421-anti-CD69, PE-anti-FoxP3, PE/Cy7-anti-Helios, PE-anti-IL-10, APC-anti-IL17A (all BioLegend), and APC-anti-RORγT, eFluor450-anti-IFNγ (Invitrogen). For intracellular cytokine and transcription factor staining, the mouse FoxP3 buffer set and the Cytofix/Cytoperm kit (BD) were used. Samples were analyzed on an LSR Fortessa (BD) and analyzed using FlowJo (BD).

### Organ processing for flow cytometry analysis

Single-cell suspensions were prepared from spleen, mesenteric lymph nodes, and Peyer’s patches by mechanical disruption through 70 μm cell strainers. Colonic lamina propria lymphocytes were isolated from the large intestine by removing fatty tissue and fecal contents, opening the intestine longitudinally, cutting it into small pieces, and processing the tissue using the gentle MACS Octo Dissociator protocol (Miltenyi). For surface marker detection, cells were first stained with viability dye (Zombie UV, Biolegend or eFluor™ 506, Invitrogen) and blocked with Fc blocker (Miltenyi). Staining was performed using the following antibodies: PerCP/Cy5.5-anti-TCR Vβ11, PE/Cy7-anti D3e, PerCP/Cy5.5 or AF700-anti CD4, PE/Dazzle or BV421-anti CD8a, FITC-anti CD45, APC/Cyanine7-anti CD11b, BV421-anti CD69, APC/Cy7-anti CD25, PE/Cy7-anti B220 (all Biolegend). For intracellular cytokine staining, cells were activated with 20 ng/ml PMA, 1 μg/ml ionomycin, and 5 mg/ml brefeldin A for 4 h Afterwards, cells were fixed with 4% PFA, permeabilized and stained with: APC-anti IL17A, PE-anti IL10, PE-anti FOXP3, PE/Cy7-anti Helios, eFluor450-anti-IFNγ, and APC-anti RORγt (all Biolegend, Invitrogen). Cells were acquired on an LSR Fortessa and analyzed using FlowJo software.

### Proliferation assay

For the proliferation assay, 1.5 × 10^5^ splenocytes were cultured in the presence of various concentrations of MOG_35-55_ or OVA_323-339_ (Anaspec) as indicated. Proliferative response was measured by the incorporation of [3 H]-thymidine (1 mCi/well) during the last 16 h of a 72 h culture period. Proliferation assays were performed at least in triplicates.

### Immunofluorescence and Histology

Mice were deeply anesthetized with sodium pentobarbital (100 mg/kg) and transcardially perfused with ice-cold PBS followed by 4% PFA. Organs were extracted and post-fixed in PFA for 24 h. After post-fixation, samples were cryoprotected in 30% sucrose in PBS for 48 h at 4 °C and embedded in optimal cutting temperature compound (Tissue-Tek). Cryosections (16 µm) were collected on superfrost slides using a CM3050S cryostat (Leica). Sections were blocked in 0.1 M PBS/0.1% Triton X-100/10% goat serum for 1 h at room temperature. Primary antibodies were incubated overnight at 4 °C. After washing in 0.1 M PBS, secondary antibodies were incubated for 2 h at room temperature. Immunofluorescence was carried out using the following primary antibodies: anti-Iba1 (rabbit polyclonal; 1:500, Wako), anti-CD3 (rat monoclonal, clone KT3; 1:100, Invitrogen), anti-MBP (rat monoclonal, clone 12; 1:200, Merck), anti-CD45R (B220, rat monoclonal, clone RA3-6B2; 1:200, Invitrogen), and anti-neurofilament (mouse monoclonal, clone SMI312; 1:1000, Biolegend). Slides were mounted with DAPI Fluoromount-G (Thermo Fisher). Image analysis was performed blinded.

For Luxol Fast Blue staining, tissue sections were sequentially washed in 70% ethanol for 2 min, followed by 95% ethanol for 2 min, and then incubated overnight at 57 °C in 0.1% luxol fast blue solution. After incubation, sections were rinsed twice in 95% ethanol. The sections then underwent multiple cycles of dipping in 0.05% lithium carbonate (in ddH₂O), rinsing in ddH₂O, washing in 70% ethanol, and a final rinse in ddH₂O. Next, the sections were stained in 0.25% cresyl echt violet solution for 5 min at 57 °C, followed by sequential dips in several changes of 95% ethanol and absolute alcohol. Finally, the sections were fixed in xylene and mounted with Eukit.

### Image acquisition and analysis

Images were acquired with either Leica TCS SP8 laser confocal (405/488/552/638 nm), Leica DM6 B (equipped with K5 camera) or DMi8 widefield (equipped with Leica DFC7000 GT camera) microscopes with 10x, 20x, 40x or 63x objectives; fluorescent confocal pictures are Z-stack images, unless stated otherwise. Images were processed using Fiji ImageJ (v2.0) and exported to vector-based software (Adobe Illustrator and Affinity Designer) for figure generation.

### Single cell RNA sequencing analysis

A single-cell suspension was obtained from mesenteric lymph nodes by gently pressing the organ through a 70 μm cell strainer. The cell pellet was resuspended, and lymphocytes were counted using a hemocytometer. Cells were loaded onto the Chromium Controller/Chromium X (10 x Genomics) for synthesis of barcoded cDNA to recover 16000 cells. Libraries were constructed from the cDNA according to the manufacturer’s guidelines and sequenced on an Illumina NovaSeq 6000 platform with 150 bp paired-end mode. Fastq files were processed with cellranger version 5.0.1 using the reference refdata-cellranger-mm10-3.0.0. DropletUtils version 1.18.1 was used for cell calling.^36^ The probability that a droplet contained a real cell was calculated using the emptyDrops() function with the parameter by.rank = 17000. Droplets with an fdr < 0.01 were called as cells for the samples “BAD3517-3a3-210601”, “BAD3517-4b3-210601”, “BAD3517-5c3-210603”, and “BAD3517-6d3-210603”. Samples “BAD3517-7e3-210603” and “BAD3517-8f3-210603” had droplets with an fdr < 0.0001 called as cells.

Initial clustering analysis was conducted using Seurat version 4.3.0 with the default pipeline.^37^ Data were normalized with SCTransform(), dimensionality was reduced using RunPCA(), and harmony embeddings were calculated using harmony version 0.1.1.^38^ Clustering was performed using RunUMAP(), FindNeighbors(), and FindClusters() with the harmony embeddings. Putative multiplets were removed using scDblFinder version 1.12.0.^39^ Adaptive outlier detection was performed using scran version 1.26.2^40^ for metrics such as the number of detected genes, counts, and fraction of mitochondrial genes. Outliers were identified as cells that deviated by more than three median absolute deviations (MAD) from the median.

Ambient RNA contamination was quantified and corrected using SoupX (version 1.6.2),^41^ by manually estimating contamination and applying correction based on a predefined set of genes (Supplemental Table Z1).

Corrections were guided by cluster annotations from the initial unsupervised clustering analysis. After correction, data were reprocessed following the same pipeline as the initial clustering analysis. Cluster-specific marker genes were identified using the findMarker() function from scran version 1.26.2.^40^ Differential gene expression analysis was performed using a pseudobulk approach with DESeq2 version 1.38.3.^42^ For each cell type cluster, raw cell counts were summed for each sample, excluding genes not detected in at least 10 cells. A pairwise comparison of Sal-MOG vs OVA was performed using the DESeq2 Wald test with an adjusted *p*-value of 0.05. Functional enrichment of differentially expressed gene sets was carried out using ClusterProfiler v4.6.0 with GO terms.^43^

The single-cell RNA transcriptome dataset has been deposited at the NCBI Gene Expression Omnibus (GEO) under accession number **GSE243972**, accessible at:

https://www.ncbi.nlm.nih.gov/geo/query/acc.cgi?acc=GSE243972, using the token: **mhybgsicddopnij**.

The supporting single-cell RNA sequencing analysis files (including UMAP embeddings, QC metrics, marker gene expression, differential expression results, and background correction genes) are available in the supplementary tables.

### SPOKE analysis

We utilized the SPOKE knowledge graph to interpret the distinct scRNA-seq signatures. SPOKE is a large-scale knowledge graph that integrates over 40 biological and medical databases, with more than 35 million nodes and 100 million semantic relationships, including connections to diseases, drugs, symptoms, and reactions.^44^ Degree-weighted path counts (DWPC)^45^^,^^46^ were computed to assess connections between genes that were significantly upregulated (adj. *p* < 0.05; log fc > 0 for MOG vs. OVA comparison) in each cluster and any SPOKE node (end node) within a path length of three (Supplementary Figure 8 A, B). The DWPCs (Supplementary Figure 8 C) were grouped by entry-end node pairs and summed to calculate DWPCpair (Supplementary Figure 8D). These pairs were then filtered to retain only the top 5% of pairs per node type. For each cluster, the DWPCpairs were weighted by the log2 fold change (log2fc) of the corresponding entry node. The weighted DWPCpairs were then grouped by end node and summed to calculate the end node score (ENS) for each cluster. ENSs were normalized against a background of random ENSs, generated from 500 permutations of the clusters' differentially expressed genes (DEGs) (Supplementary Figure 8E). To visualize the results, we extracted nodes with the highest values for each analyzed cluster and represented them in a connected sub-graph. Entry nodes (differentially expressed genes) are depicted as circles, and the highest-scoring nodes of different types are displayed inside these circles. The SPOKE network generated in this study is publicly available on **Zenodo** (DOI: https://doi.org/10.5281/zenodo.16927590). This file can be downloaded and opened directly in Cytoscape (https://cytoscape.org/) for interactive visualization and further exploration of the network structure.

### Statistical analysis

Statistical analyzes of flow cytometry and histopathological quantifications were performed using parametric tests, including the unpaired Student’s t-test for comparisons between two groups and one-way or two-way ANOVA followed by Tukey’s post hoc test for comparisons involving three groups. Multiple comparisons were adjusted using the Holm-Šídák method. For data that did not meet normality assumptions, non-parametric tests were used: the Mann-Whitney U test for two-group comparisons and the Kruskal-Wallis test followed by Dunn’s post hoc test for comparisons involving three groups. Data are presented as mean ± standard deviation (SD) or standard error of the mean (SEM). All tests were performed using two-tailed analysis. The significance threshold was set at *p* < 0.05, and *p* values were indicated as follows: *p < 0.05; **p < 0.01; ***p < 0.001. All statistical analyzes were conducted using GraphPad Prism software (version 10.3.1).

For EAE clinical scores, a repeated-measures two-way ANOVA was applied. Time (days post-immunization) was included as a within-subjects factor, and the experimental group was considered a between-subjects factor. The model assessed main effects for time and group, as well as their interaction (time x group). Significant differences between experimental groups over time are indicated by brackets, and significance was only considered when the time x group interaction was significant.

## Results

### Generation of prototypic inflammatory gut bacteria expressing a self-epitope

**Figure 1.**
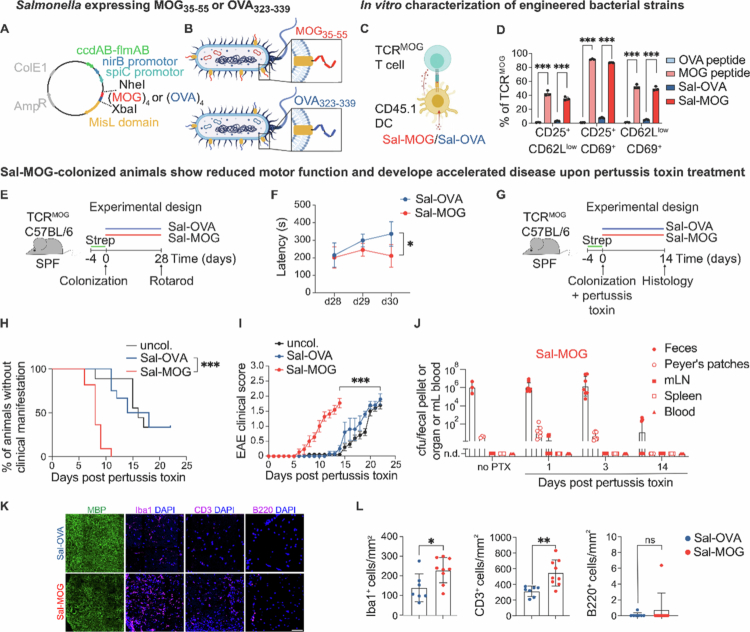
Colonization of SPF TCR^MOG^ mice with MOG_35-55_-expressing *Salmonella* leads to accelerated neuroinflammation. (A, B) Schematic overview of the generated expression system (A) and *Salmonella* strains expressing either MOG_35-55_ (Sal-MOG) or OVA_323-339_ (Sal-OVA) on their surface (B). (C) Schematic overview of the experiment shown in (D). Co-cultivation assay of TCR^MOG^ CD4^+^ T cells and *in vitro* primed CD45.1 DCs (stimulation with either peptide or heat-inactivated *Salmonella* strains). (D) Flow cytometry analysis of the resulting T cell activation. (E) Experimental set-up for the colonization of SPF housed TCR^MOG^ mice with streptomycin (Strep) pretreatment, washout and colonization period. (F) Rotarod assessment of motor performance after colonization. (G) Schematic illustration of disease induction by pertussis toxin injection upon colonization after streptomycin (Strep) pretreatment and washout. (H) Percentage of mice showing no clinical symptoms during observation period. (I) EAE scores of Sal-MOG and Sal-OVA treated mice together with uncolonized (uncol.) TCR^MOG^ mice receiving pertussis toxin only. (J) Bacterial burden (presented as colony-forming units, cfu) of Sal-MOG colonized mice in feces, Peyer’s patches, mLN, spleen and blood at indicated timepoints assessed by plating on LB agar. “No PTX” refers to mice which have been analyzed on day 1 post colonization but have not received any pertussis toxin administration. (K) Representative histopathological assessment of the spinal cord (dorsal fiber tracts) for Sal-MOG versus -OVA treated EAE mice 14 days post colonization/pertussis toxin administration. Scale bar: 50 µm. (L) Quantification of Iba1^+^, CD3^+^ and B220^+^ cells between Sal-MOG vs. -OVA treated EAE mice. (D) shows example of three independent experiments. (F) shows example of three independent experiments with *n* = 5 mice/group. (H, I) show pooled analysis of three independent experiments with at least *n* = 3 mice per group/experiment. (J) Pooled analysis of two experiments with at least *n* = 3 mice per group/experiment. (L) Pooled analysis of at least two independent experiments with at least 3 mice per group/experiment. (D) Multiple unpaired *t*-test, Holm-Šídák method. (F) Repeated-measures two-way ANOVA with Bonferroni’s multiple comparisons test. (I) Repeated-measures two-way ANOVA between Sal-MOG vs. Sal-OVA between day 0-14. (H) Log-rank test was performed between Sal-MOG and Sal-OVA mice between day 0-14. (L) Unpaired *t*-test. (D, F, J, L) Mean ± SD, (H) Mean ± SEM. (J, L) Each dot represents one mouse. ns = not significant; *p < 0.05; **p < 0.01; ***p < 0.001. uncol. = uncolonized, MOG = myelin oligodendrocyte glycoprotein, OVA = ovalbumin, SPF = specific pathogen-free, EAE = experimental autoimmune encephalitis. Related to Supplementary Figure 1 and 2.

We engineered the attenuated *Salmonella* Δ*aroA* strain SL7207^25^ as a model system for a pro-inflammatory gut bacterium to express an encephalitogenic peptide of MOG (MOG_35-55_) or ovalbumin (OVA_323-339_; OT-II epitope) as an isogenic control. SL7207 is restricted to the gut and its immune compartments,^47^ allowing local introduction of the target antigen. To enhance immunogenicity, we fused an antigen concatemer of the immunodominant MHC class II (I-A^b^) restricted MOG_35-55_ and OVA_323-339_ epitopes to the well-characterized MisL autotransporter enabling surface expression^23^^,^^24^ (Figure 1A,B; Supplementary Figure 1A,B).

### Engineered strains of *Salmonella* stimulate myelin-specific T cells *in vitro*

We took advantage of an existing murine model of autoimmune neuroinflammation to derive TCR^MOG^-transgenic 2D2 T cells, while T cells from TCR^OVA^-transgenic OT-II mice served as controls. To validate the immunogenic potential of the engineered strains (hereafter, Sal-MOG or Sal-OVA), we employed an *in vitro* co-culture system exposing T cells to heat-inactivated Sal-MOG- or -OVA-primed DCs (Figure 1C). MOG-reactive CD4^+^ T cells were only activated by the MOG peptide or MOG-expressing bacteria, whereas OVA-reactive T cells were only activated by the respective OVA peptide or OVA-expressing bacteria (Figure 1D; Supplementary Figure 1C, D). T cell differentiation and cytokine secretion remained unaltered between T cells stimulated with Sal-MOG or -OVA (Supplementary Figure 1E–H). Taken together, we have generated two isogenic bacterial strains, differing only in the surface-exposed antigen. MOG_35-55_-, but not OVA_323-339_-expressing bacteria were able to activate MOG-reactive T cells *in vitro*.

### Colonization with MOG_35-55__-_expressing bacteria leads to reduced motor function and accelerates disease

The encephalitogenic potential of Sal-MOG was tested *in vivo*. To that end, we colonized specific-pathogen-free (SPF) housed TCR^MOG^ mice (Figure 1E). As expected,^47^ colonization with Sal-OVA or Sal-MOG did not lead to symptomatic infection. However, Sal-MOG colonized mice showed deficits in motor function compared to Sal-OVA colonized mice as assessed by rotarod (Figure 1F), which has been shown to correlate with inflammatory lesions and demyelination of motor nerves in the spinal cord.^48^ This prompted us to test whether increased blood-brain barrier permeability induced by pertussis toxin^49^ would aggravate disease (Figure 1G). Indeed, we observed clinical disease manifestation in all mice colonized with Sal-MOG within 6-7 days after pertussis toxin administration (Figure 1H,I). In agreement with previous reports in uncolonized TCR^MOG^ mice,^32^ pertussis toxin injections into Sal-OVA colonized mice also induced disease, albeit with a delayed disease onset compared to Sal-MOG colonized mice (Figure 1H,I).

To assess potential systemic bacterial translocation, we quantified Sal-MOG bacteria in feces, local (Peyer’s patches, mesenteric lymph node (mLN) and systemic (spleen, blood) immune compartments prior and after pertussis toxin administration (Figure 1J). As anticipated, we detected bacteria in feces and, to a lower extent, in Peyer’s patches.^47^ We observed colonies in the mLN 24 h post pertussis toxin administration only in 2 out of 9 mice but in none of the analyzed spleens or blood samples (Figure 1J).

Histopathological assessment of the CNS specimens at peak disease confirmed inflammatory infiltrates along the dorsal fiber tracts of the spinal cord of Sal-MOG-colonized mice (Figure 1K,L). In detail, we observed a marked increase in CD3^+^ T cells and Iba1^+^ myeloid cells within the affected tissue, indicative of significant T cell infiltration, recruitment of tissue-infiltrating myeloid cells, and potentially local microglial proliferation. These findings align with hallmark features of robust neuroinflammatory processes involving both adaptive and innate immune cell populations. Conversely, we did not detect relevant B220^+^ B cell tissue infiltrates accompanied by preserved axonal numbers at this acute disease stage as assessed by SMI312 staining (Figure 1L; Supplementary Figure 2A, B).

We thus conclude that pro-inflammatory bacteria presenting a self-epitope in the local immune compartments of the gut can accelerate neuroinflammation facilitated by blood-brain barrier perturbation.

### Mono-colonization with MOG_35-55__-_expressing *Salmonella* induces spontaneous CNS inflammation and accelerates disease

**Figure 2.**
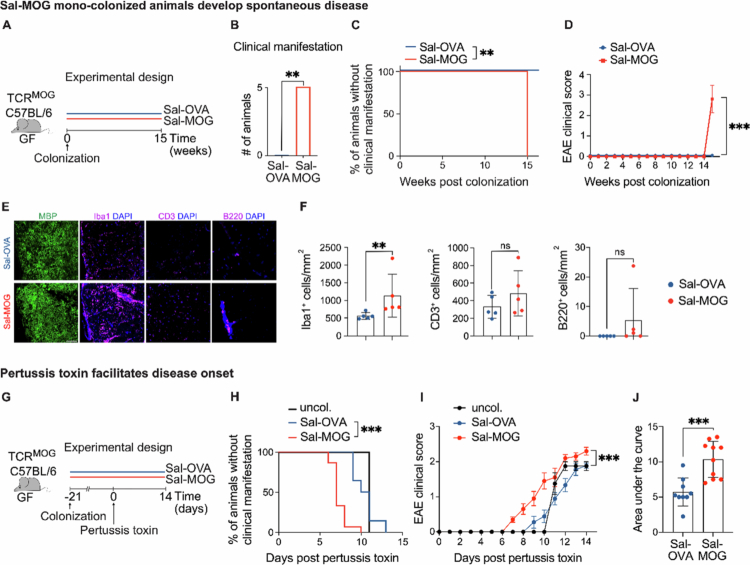
Mono-colonization of GF TCR^MOG^ mice with MOG_35-55_-expressing *Salmonella* leads to neuroinflammation. (A) Experimental set-up of the experiment shown in (B-F). Mono-colonization of GF TCR^MOG^ mice with Sal-OVA or -MOG. (B) Number of mice showing a clinical EAE manifestation in Sal-MOG vs. -OVA colonized mice at day 15. (C) Percentage of mice without clinical manifestation during the observation time. (D) EAE scores of Sal-MOG vs. -OVA colonized mice. (E) Representative histopathological analysis of the spinal cord (dorsal fiber tracts) from mono-colonized mice. Scale bar: 50 µm. (F) Quantification of (E) Iba1^+^, CD3^+^ and B220^+^ cells in the spinal cord of Sal-OVA vs -MOG colonized mice. (G) Experimental set-up shown in (H-J). Mono-colonization of GF TCR^MOG^ mice with Sal-OVA or-MOG and pertussis toxin treatment 21 days post colonization. (H) Percentage of Sal-MOG, Sal-OVA and uncolonized (pertussis toxin only) mice without clinical manifestation during the observation time. (I) EAE scores of uncolonized vs. Sal-MOG vs. -OVA colonized mice. (J) Overall disease severity as determined by the area under the curve analysis between Sal-MOG and Sal-OVA. (B-F) show example of at least two independent experiments with *n* = 5/group. (H-J) Pooled analysis of at least two independent experiments with at least *n* = 4 mice per group/experiment. (B) Fisher’s exact test. (C) Log-rank test. (D) Repeated-measures two-way ANOVA. (F) Unpaired *t*-test. (H) Log-rank test was performed between Sal-MOG vs. -OVA mice. (I) Repeated-measures two-way ANOVA between Sal-MOG vs. Sal-OVA mice. (J) Unpaired *t*-test. (D, I) Mean ± SEM. (F, J) Mean ± SD. (F, J) Each dot represents one mouse. ns = not significant; *p < 0.05; **p < 0.01; ***p < 0.001. uncol. = uncolonized. Sal = *Salmonella,* MOG = myelin oligodendrocyte glycoprotein, OVA = ovalbumin, GF = germ-free, EAE = experimental autoimmune encephalitis. Related to Supplementary Figure 3.

To precisely delineate the antigen-specific effects of the engineered bacterial strains, we rederived TCR^MOG^-transgenic mice under GF conditions. Mono-colonization of transgenic GF mice with Sal-MOG but not -OVA resulted in spontaneous development of clinical disease 15 weeks post-inoculation (Figure 2A−D). To additionally assess subclinical disease, the spinal cord of all mice was harvested immediately after disease onset in Sal-MOG colonized mice. Histopathological analysis confirmed a marked increase of Iba1^+^ microglia/macrophages in the spinal cord of Sal-MOG but not -OVA colonized mice (Figure 2E,F). At this disease stage, we did not observe differential infiltration of adaptive immune cells in comparison to Sal-OVA colonized mice (Figure 2E,F), neither did we detect any differences in SMI312^+^ axonal numbers between Sal-OVA- vs. MOG colonized mice (Supplementary Figure 3A). In line with our SPF model, administration of pertussis toxin into GF TCR^MOG^ mice mono-colonized with Sal-MOG accelerated disease progression (Figure 2G−I; Supplementary Figure 3B) and exacerbated the overall disease course in comparison to Sal-OVA colonized mice (Figure 2J). Remarkably, mono-colonization of WT C57BL/6 mice with Sal-MOG also exacerbated experimental autoimmune encephalomyelitis (EAE) (Supplementary Figure 4A-C), despite the low proportion of MOG-specific T cells in the endogenous repertoire. This was further supported by increased Iba1^+^ inflammatory infiltrates in the spinal cords of WT Sal-MOG, but not WT Sal-OVA, colonized mice (Supplementary Figure 4D).

Taken together, our observations indicate that the local presentation of a self-antigen in the gut exacerbates neuroinflammation and neurological disease.

### Antigenic activation of autoreactive T lymphocytes in the mesenteric lymph node

**Figure 3.**
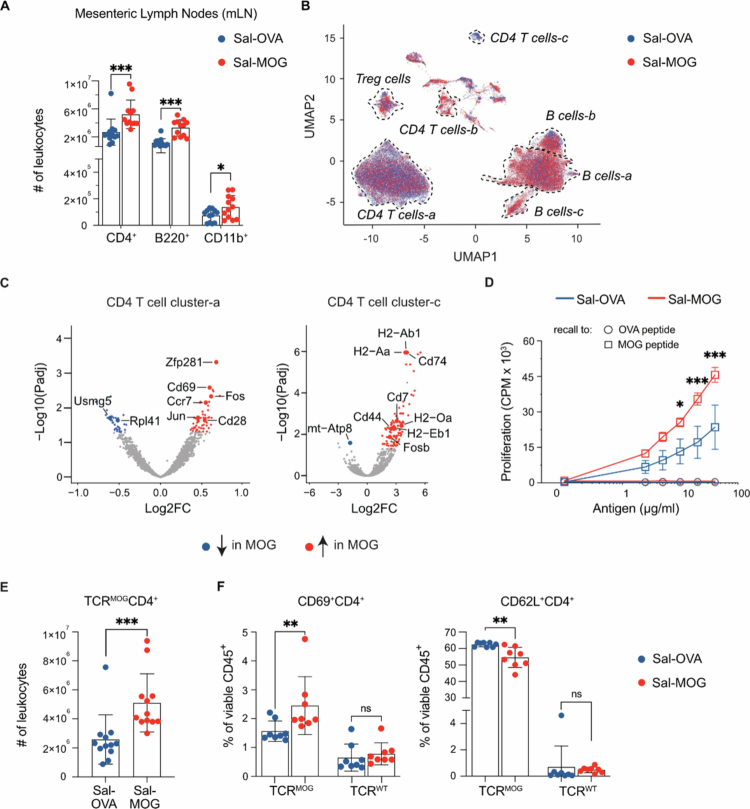
Mono-colonization of GF TCR^MOG^ mice with MOG_35-55_-expressing *Salmonella* leads to activation of MOG-reactive T cells. (A) Quantitative flow cytometry analysis of immune cell subsets in mesenteric lymph nodes (mLN) of Sal-MOG vs. -OVA colonized mice. (B) RNA-seq analysis of mLN from Sal-MOG and -OVA mono-colonized TCR^MOG^ mice; shown is the uniform manifold approximation and projection (UMAP) visualization colored by experimental group; dotted lines outline T- and B-cell type annotations which were based on expression and specificity of genes depicted in Figure S6B. (C) Volcano plots showing differentially expressed genes in CD4 T cell cluster –a (left) and-c (right), respectively, between Sal-MOG vs. Sal-OVA mice. (D) *Ex vivo* proliferation analysis of splenocytes from mono-colonized TCR^MOG^ mice three weeks post colonization. [3 H]-thymidine incorporation (counts per minute, CPM) was measured upon stimulation with either MOG_35-55_- or OVA_323-339_-peptide. (E) Quantitative flow cytometry analysis of TCR^MOG^ CD4^+^ T cells in mLN of Sal-MOG vs. Sal-OVA mono-colonized mice. (F) Percentage of CD69 (left) and CD62L (right) expressing TCR^MOG^ and TCR^WT^ CD4^+^ T cells in mLN of Sal-MOG vs. Sal-OVA mice measured by flow cytometry. Experiments in (A, E, F) show three (A, E) or two (F) independent experiments with *n* = 4 mice per group/experiment. (C) Analysis was performed with *n* = 3 mice per group. Red and blue dots represent dysregulated genes according to a cutoff of Padj = 0.05. (D) *n* = 4 mice per group. (A, E, F) Unpaired *t*-test; mean ± SD. Each dot represents one mouse. (D) Ordinary two-way ANOVA followed by Tukey’s multiple comparisons test (significant differences are depicted for MOG peptide only); mean ± SEM. ns = not significant. *p < 0.05; **p < 0.01; ***p < 0.001. Sal = *Salmonella,* MOG = myelin oligodendrocyte glycoprotein, OVA = ovalbumin; Padj = adjusted *P* value, FC = fold change. Related to Supplementary Figure 5 and 6 and Table Z4.

To identify the site of immune cell activation, we analyzed local (lamina propria, Peyer’s patches, mLN) and systemic (spleen) immune compartments in GF TCR^MOG^ mice 3 weeks after mono-colonization with Sal-MOG or -OVA, and prior to disease induction. We observed an expansion/infiltration of CD4^+^, B220^+^ and CD11b^+^ immune cells in the mLN (Figure 3A; Supplementary Figure 5 A) but not in the lamina propria, Peyer’s patches or the spleen of Sal-MOG mice (Supplementary Figure 5 A,B).

To elucidate molecular processes underlying local immune activation, we performed single-cell RNA sequencing (scRNA-seq) analysis of mLNs from GF TCR^MOG^ mice, mono-colonized with Sal-MOG or Sal-OVA (Figure 3A,B; Supplementary Figure 4A-C; Tables Z2-4). The highest numbers of differentially expressed genes (DEGs) were identified in CD4^+^ T cells and B cells (Figure 3B; Supplementary Figure 6A-C). Within the CD4^+^ T cell compartment (Figure 3C; Table Z4) two distinct clusters (CD4^+^ T cell clusters -a and -c) exhibited the most changes upon Sal-MOG mono-colonization (Supplementary Figure 6B), both characterized by genes associated with activated memory CD4^+^ T cells (Figure 3C). In CD4^+^ T cell cluster-a, Sal-MOG mono-colonization led to increased expression of antigen-specific activation genes, including *Cd69*^50^*, Fos* and *Jun*^51^ (Figure 3C). Furthermore, *Cd28*^52^ and *Ccr7*^53^, both essential for peripheral CD4^+^ T cell priming and expansion during EAE development, were markedly upregulated in Sal-MOG mono-colonized mice. In CD4^+^ T cell cluster-c, increased *Cd44*^54^ expression, indicative of antigen-experienced effector and memory T cells, and elevated *Cd74*^55^, which encodes a receptor for the cytokine MIF with roles in inflammation and immune activation, further point to local, antigen-specific T cell activation in the mLN.

Consistent with the transcriptomic data, MOG-reactive CD4^+^ T cells from Sal-MOG mono-colonized mice exhibited enhanced proliferative capacity at both 3 weeks post-colonization (Figure 3D) and at peak disease (Supplementary Figure 6D). Flow cytometry analysis revealed a significant increase in the number of MOG-reactive CD4^+^ T cells in the mLN of Sal-MOG mono-colonized mice (Figure 3E), with no changes observed in the lamina propria, Peyer’s patches, or spleen (Supplementary Figure 6E). Importantly, TCR^MOG^ but not TCR^WT^ CD4^+^ T cells in the mLN of Sal-MOG, but not Sal-OVA mono-colonized mice displayed elevated levels of the early activation marker CD69 and decreased CD62L expression (Figure 3F), indicative of antigen experience and effector/memory differentiation.^56^ Additionally, the percentage of CD69-expressing TCR^MOG^ CD4^+^ T cells was significantly higher in the spleen of Sal-MOG mono-colonized mice (Supplementary Figure 6F), likely due to the egress of activated MOG-specific T cells from the mLN into systemic circulation. No significant differences were observed in the lamina propria or Peyer’s patches upon Sal-MOG colonization (Supplementary Figure 6F).

Analysis of cytokine profiles across immune compartments revealed that, overall, MOG-reactive CD4^+^ T cell differentiation patterns were similar between groups (Supplementary Figure 7A, B). However, there was an increased percentage of TCR^MOG^ CD4^+^ T cells expressing IL17 in the lamina propria and a trend toward more IFN-γ/IL17 double-positive cells in the mLNs of Sal-MOG but not Sal-OVA colonized mice (Supplementary Figure 7B). These findings suggest that while the overall differentiation program remains comparable, Sal-MOG colonization subtly skews pro-inflammatory cytokine production in gut-related immune compartments.

To gain a deeper mechanistic understanding of the trajectories of locally activated autoreactive immune cells in the intestinal immune compartment, we employed SPOKE, a comprehensive biomedical knowledge graph that integrates data from over 40 public databases.^44^ Due to the nature of linking related information from multiple sources, SPOKE analyzes can highlight functionally related concepts via multiple “hops” within the network. By mapping our scRNA-seq transcriptomic data to SPOKE, we analyzed the connections between upregulated genes and key biological concepts, such as diseases and cellular processes, with a focus on significant gene expression changes (Supplementary Figure 8; Supplementary Figure 9). SPOKE analysis revealed distinct patterns in CD4^+^ T cell clusters (Supplementary Figure 9A). For CD4⁺ T cell cluster-a, we observed links to several CNS-related features, including neurological diseases, anatomical regions, and symptoms, suggesting a potential link between gut-driven immune activation and CNS pathology. In contrast, the differentially expressed genes in CD4⁺ T cell cluster-c were associated with a broader array of concepts such as metabolic pathways, gut barrier integrity, and transcriptional regulation (Supplementary Figure 9A). In summary, colonization with gut bacteria expressing self-epitopes leads to antigen-specific activation of autoreactive T cells in the mLN with implications for neuroinflammation.

### Activation of B cells leads to autoantibody production

Interestingly, we also observed a higher number of differentially expressed genes in the B cell compartment of Sal-MOG mono-colonized mice, particularly in clusters-a and -b (Supplementary Figure 10 A). Both clusters exhibit signatures of mature B cells, with cluster-b displaying a more activated phenotype (Supplementary Figure 10 A). When comparing Sal-MOG to Sal-OVA colonized mice, key activation markers, *Fos, Jun*^57^*, Stat1*^58^, and *Stat3*^59^, were upregulated (Supplementary Figure 10 A; Table Z4), indicating robust antigen-driven activation. Moreover, the downregulation of the pre-BCR transcript *Vpreb3*, alongside the upregulation of *Ighg3* in B cell cluster-a, suggests a shift from immature to class-switched, antigen-experienced B cells.^60^ In line with these findings, we detected MOG_35-55_-reactive antibodies in the serum of Sal-MOG but not -OVA colonized mice (Supplementary Figure 10B). Since no relevant B cell tissue infiltration has been observed in Sal-MOG colonized mice (Figures 1L and 2F), we assume these antibodies are produced in the periphery. These findings highlight that gut-specific MOG antigen exposure drives B cell activation and maturation within the mLN.

SPOKE analysis of B cell cluster-a revealed enrichment in classical and gut-associated immune activation pathways, highlighting concepts such as immunodeficiency and Peyer’s patches (Supplementary Figure 9B). B cell cluster-b pointed to concepts related to the gut-brain axis, such as axon regeneration, spinal cord segments, and colon inflammation (Supplementary Figure 9B). These results reinforce our experimental findings, emphasizing the link between the local autoreactive immune response in the gut and neuroinflammation.

### Disease induction by gut-colonizing MOG-expressing bacteria is not limited to *Salmonella*

**Figure 4.**
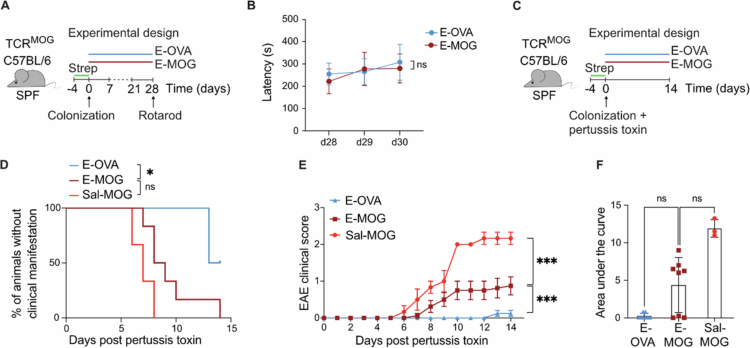
MOG_35-55_ - expressing *E. coli* induce neuroinflammation in SPF TCR^MOG^ mice. (A) Schematic overview of the experiment shown in (B). Colonization of SPF-housed TCR^MOG^ C57BL/6 mice with *E. coli* strains expressing either MOG_35-55_ (E-MOG) or OVA_323-339_ (E-OVA) on their surface. (B) Rotarod assessment of motor performance after colonization as depicted in (A). (C) Experimental set-up of the experiment shown in (D-F). Colonization of SPF-housed TCR^MOG^ C57BL/6 mice—after Streptomycin (Strep) pretreatment and washout—with E-OVA or -MOG in comparison to Sal-MOG after pertussis toxin administration. (D) Percentage of mice without clinical manifestation during the observation time. (E) EAE clinical scores of E-MOG versus -OVA colonized mice in comparison to Sal-MOG. (F) Overall disease severity as determined by the area under the curve analysis. Experiment in (B) shows an example of at least two independent experiments using at least *n* = 4 mice per group. (D-F) Data was pooled from two independent experiments with *n* = 4 mice per group/experiment; except from Sal-MOG: *n* = 3 mice per group. (B) Repeated-measures two-way ANOVA. (D) Log-rank test. (E) Repeated-measures two-way ANOVA. (F) Kruskal-Wallis test followed by Dunn’s post hoc test. Each triangle/square/dot represents one mouse. (B, F) Mean ± SD. (E) Mean ± SEM. ns = not significant; *p < 0.05; **p < 0.01; ***p < 0.001. Sal = *Salmonella,* MOG = myelin oligodendrocyte glycoprotein, OVA = ovalbumin, SPF = specific pathogen free, EAE = experimental autoimmune encephalitis. Related to Supplementary Figure 11.

To evaluate whether disease induction is restricted to the prototypic proinflammatory *Salmonella* strain, we engineered the commensal *E. coli* strain MP1^28^ to express MOG_35-55_ or OVA_323-339_ (Supplementary Figure 11A-C; hereafter: E-MOG and E-OVA). In SPF TCR^MOG^ mice, rotarod performance did not differ between the E-MOG and E-OVA groups (Figure 4A,B). However, when pertussis toxin was administered, colonization with E-MOG induced neurological disease in comparison to E-OVA colonized mice (Figure 4C–E), albeit with a significantly milder disease course compared to Sal-MOG colonization (Figure 4E). Similar differences were also observed in mono-colonized GF TCR^MOG^ mice (Supplementary Figure 11E–G). Although there was no significant variation in colonization efficiency (Supplementary Figure 11 K), the factors underlying the differences in disease course between E‑MOG and Sal‑MOG strains may include variations in local MOG expression levels.

When analyzing the mLN at peak disease, E-MOG mono-colonized GF mice did show higher expression levels of CD69 in TCR^MOG^ CD4^+^ T cells (Supplementary Figure 11 H). In contrast, no increase in B220⁺ lymphocytes was observed in the mLN (Supplementary Figure 11I). Furthermore, the absence of detectable MOG_35-55-_reactive antibodies in E-MOG colonized mice (Supplementary Figure 11 J, compared to Supplementary Figure 10B) supports reduced activation of B cells. Thus, we conclude that the disease induction is not specific for MOG-expressing *Salmonella*.

## Discussion

In this study, we present a proof-of-concept experimental model that delineates mucosal triggers contributing to systemic autoimmunity (Supplementary Figure 12). Our findings demonstrate that gut-restricted bacteria expressing a self-antigen can initiate local immune activation leading to subsequent CNS inflammation, a process that is influenced by the bacterial context and requires blood-brain barrier disruption.

Local antigen-specific involvement of gut bacteria has been shown in murine colitis models.^61^ However, proof-of-concept studies for their direct influence on distal tissues in the context of autoimmunity have not been reported so far. Previous studies have provided mostly correlative evidence that associates gut bacteria containing potential molecular mimics with the development of autoimmunity,^12^^,^^14^^,^^16^^,^^62^ including MS^15^^,^^17^ and related diseases.^63^ Notably, *Acinetobacter* and *Akkermansia*, in particular, have been linked to MS,^7^^,^^8^^,^^12^^,^^64^ and exhibit potential molecular mimicry with autoimmune targets^17^^,^^65^ together with immunomodulatory properties in this disease context.^8^^,^^66^ Along these lines, encephalitogenic Th_17_ cells have been observed to migrate to and proliferate in the mouse gut before making their way to the CNS, suggesting the microbiome’s involvement in antigenic activation.^67^^,^^68^ Additionally, bacterial toxins have been found to facilitate overcoming CNS immune privilege in MS.^69^ Furthermore, the coordinated activity of microorganisms that express mimicry antigen and those that enhance pro-inflammatory T cell responses have been shown to drive autoimmune neuroinflammation.^13^

As an extension of the molecular mimicry hypothesis, existing studies have used genetically modified microbes expressing cognate myelin antigens and demonstrated that these microbes confer protection from EAE. Maassen et al. showed that *Lactobacillus casei* expressing an MBP epitope induced regulatory responses and ameliorated EAE.^18^ Similarly, Buerth et al. reported that *Candida utilis* expressing MOG_35-55_ conferred protection linked to increased Treg frequencies and reduced inflammatory cytokine production.^19^ These findings support the notion that when self-antigens are presented under non-inflammatory conditions, they primarily induce tolerance towards administered antigens. Indeed, the concept of oral tolerance, whereby mucosal exposure to self-antigen induces systemic immune hyporesponsiveness, has been well established in EAE models.^70-72^ Similar to bacterially expressed myelin antigens, feeding myelin proteins or peptides typically induces regulatory T cell responses or anergy, preventing CNS autoimmunity.^70^^,^^71^ However, it is important to note that gut microbes differ vastly in their ability to stimulate immune responses. For instance, a prominent human symbiont, *Bacteroides fragilis,* induces regulatory immune responses,^73^ whereas a segmented filamentous bacterium induces Th17 immune responses and promotes EAE development.^74^ Beyond these examples, some microbes can synergistically work together and enhance EAE severity under certain conditions.^13^

In this study, we employed an Δ*aroA*-attenuated *Salmonella* strain, which induces complex immune responses that include humoral and cellular immune responses, to express a myelin antigen. We hypothesized that the presentation of myelin antigen in the context of an inflammatory setting, as opposed to previous studies, would shift the immunological outcome from tolerance to pathogenicity by allowing priming of encephalitogenic T cells. Indeed, we observed a rapid EAE induction and enhanced EAE severity in mice colonized with MOG-peptide expressing *Salmonella* bacterium. Our results are in line with several studies, which suggest that local inflammation is necessary to overcome peripheral tolerance and activate autoreactive T cells.^13^^,^^75^^,^^76^ The divergence in outcomes between our study and oral tolerance models underscores the importance of microbial context, specifically, the immunogenic properties of the antigen-presenting microbe, in shaping the nature of antigen-specific immune responses in the gut.

The local activation of autoreactive T cells requires presentation of antigen by antigen-presenting cells (APCs) such as DCs within the gut-associated lymphoid compartments. We observed significant changes in the T cell activation status in the mLNs, although other gut-associated compartments may also contribute to the T cell activation. Moreover, it is likely that other APC types, such as macrophages or B cells, may also contribute to antigen presentation in the gut. Given the heterogeneity and tissue-specific specialization of APCs in the gut, further studies will be required to dissect their respective roles in promoting or regulating autoreactive T cell responses.

In addition to local activation of autoreactive immune cells in the gut compartment, a “leaky” gut with subsequent microbial translocation is discussed as a potential pathomechanism leading to systemic immune activation in autoimmune diseases,^75^^,^^77^ including MS.^78^^,^^79^ In our model, we do not observe bacterial translocation beyond the gut immune compartment, supporting local activation and migration of gut-originating autoreactive immune cells into the CNS. It is conceivable that additional bacterial translocation to the systemic circulation may enhance a locally triggered immune response and help to break immune self-tolerance mechanisms.

Collectively, our findings indicate three pivotal roles of gut bacteria in autoimmune neuroinflammation: 1. Antigen-specific triggering of autoreactive immune cells by molecular mimicry. 2. Modulation of the disease by the inflammatory milieu. 3. Facilitation of immune cell entry into the CNS. Diverging from recent investigations that have focused primarily on the influence of the inflammatory microbial milieu on autoimmunity,^7^^,^^8^^,^^11^ our research highlights the close interplay between antigen-specific and antigen-unspecific microbiota-immune interactions in the context of neuroinflammation.

Our study has several limitations: While we confirmed the effect of the self-epitope expressing bacteria also in a WT EAE model, we are potentially overestimating the immunogenic capacity of our system by harnessing a transgenic mouse model targeting a self-antigen^32^ and enhancing immunogenicity by surface expression of antigen concatemers. Moreover, although our analysis identified antigen-specific T cell activation in the mLN after bacterial colonization, we cannot exclude that priming may also occur at earlier or intermediate time points or in additional gut-associated sites. Furthermore, as the actual myelin autoantigens targeted in patients with autoimmune neuroinflammation remain largely unknown,^80^ future research should focus on identifying the bacterial taxa and antigens involved in disease initiation, also taking into account HLA-restriction.^15^^,^^17^ Lastly, other mucosal surfaces besides the gut might also serve as primary activation sites of autoantigen-specific immune cells^81^ and the exact migration route from peripheral tissue into the CNS needs to be further investigated.

Our research carries broader implications for the development of microbiome-based therapeutic approaches. Importantly, these findings pose a caveat in regard to autoimmune side effects as a considerable risk factor when eliciting favorable immune responses by engineered bacteria as cancer therapeutics.^82^ At the same time, our findings open novel avenues for the application of recently described strategies aiming at the targeted removal of undesired bacterial species.^3-5^ Finally, given the importance of the bacterial context in which the antigen is presented, it is tempting to speculate about designing commensal-based antigen-specific tolerogenic vaccines^83^ for the treatment of autoimmune diseases.

## Supplementary Material

Supplementary Material Supplemental_Tables

Supplementary Material Supp_figs_1-6.docx

Supplementary Material Supp_Fig_7-12.docx

Supplementary Material Siewert_Berve_Supporting_Information.docx

## Data Availability

The single-cell RNA transcriptome dataset generated in this study has been deposited in the NCBI Gene Expression Omnibus (GEO) under accession number **GSE243972** and is accessible at: https://www.ncbi.nlm.nih.gov/geo/query/acc.cgi?acc=GSE243972, using the token: **mhybgsicddopnij**. https://zenodo.org/records/15322843?preview=1&token=eyJhbGciOiJIUzUxMiJ9.eyJpZCI6ImQzY2VkY2M5LWVlZWUtNGI5MS04OTc2LTE1OTE3NmIwYjUwNiIsImRhdGEiOnt9LCJyYW5kb20iOiIzNTQ2OGZiMmY4NWE1YjAzYWQ0Y2ViOTIyZTIzZjE5NyJ9.KvqHrIXfo-fAv1-irVC32OgK_ee34XtNhTi60e9Z1hEjzyrP9qme2kvItPcsJGdhzOCOBHi61Cx9ef-OUekCxg. These datasets will be made publicly available under **a****CC-BY 4.0 license** upon publication.
