# Supplementary material for: Antigen-specific activation of gut immune cells drives autoimmune neuroinflammation
Source: Gut Microbes. 2025 Dec 24;18(1):2601430. doi: 10.1080/19490976.2025.2601430 (PMC12962552; doi:10.1080/19490976.2025.2601430)
Supplement: Supplementary Material — Supp_figs_1-6.docx [file KGMI_A_2601430_SM2396.pdf]

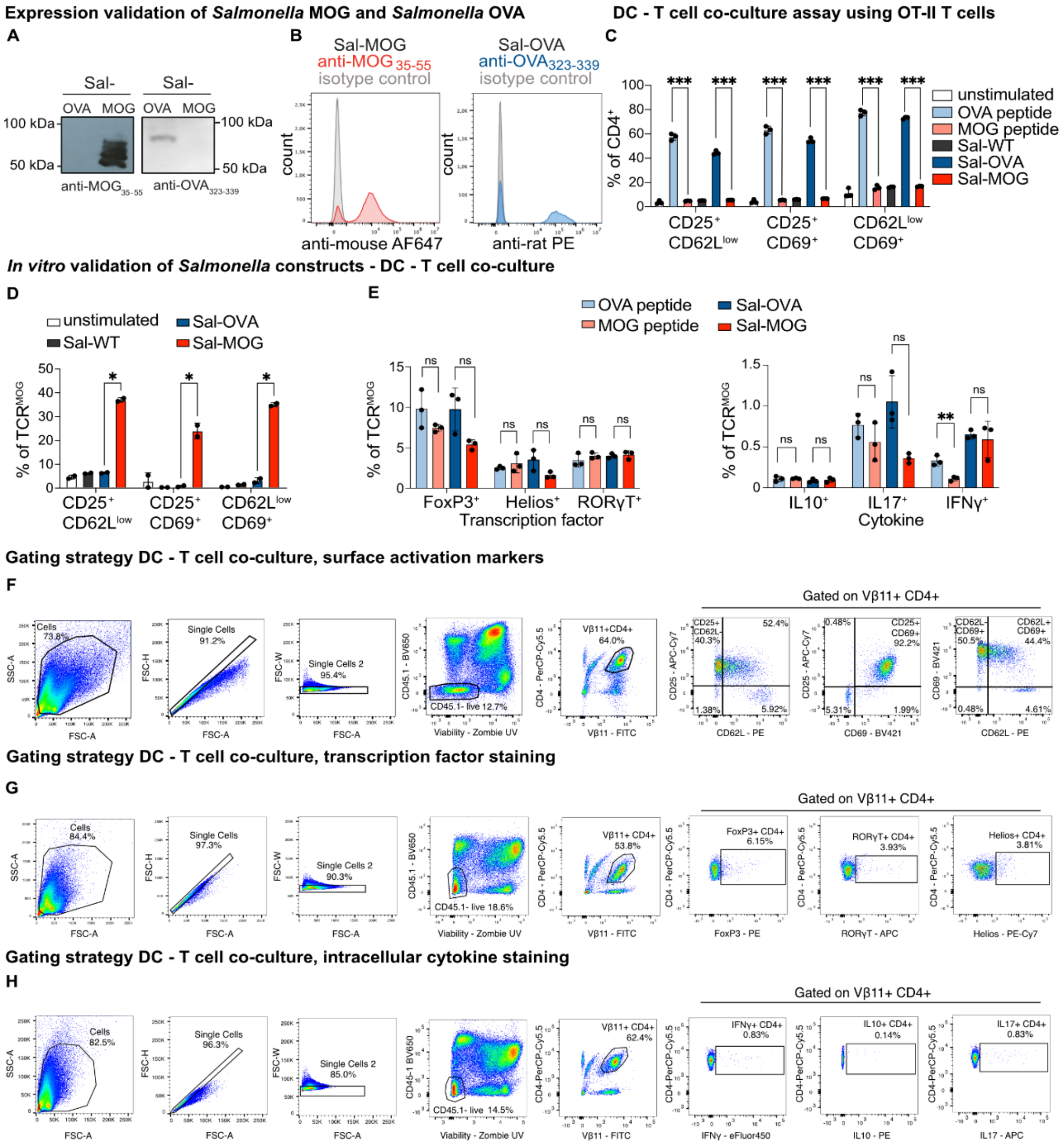
Expression validation of Salmonella MOG and Salmonella OVA
A
Sal-
Sal-
OVA MOG OVA MOG
100 kDa
50 kDa
100 kDa
50 kDa
anti-MOG35-55
anti-OVA323-339
B
Sal-MOG
anti-MOG 35-55
isotype control
Sal-OVA
anti-OVA323-339
isotype control
count
anti-mouse AF647
anti-rat PE
DC - T cell co-culture assay using OT-II T cells
C
% of CD4+
unstimulated
OVA peptide
MOG peptide
Sal-WT
Sal-OVA
Sal-MOG
CD25+ CD62Llow
CD25+ CD69+
CD62Llow CD69+
In vitro validation of Salmonella constructs - DC - T cell co-culture
D
unstimulated
Sal-OVA
Sal-WT
Sal-MOG
% of TCRMOG
CD25+ CD62Llow
CD25+ CD69+
CD62Llow CD69+
E
OVA peptide
Sal-OVA
MOG peptide
Sal-MOG
% of TCRMOG
FoxP3+
Helios+
RORγT+
Transcription factor
IL10+
IL17+
IFNγ+
Cytokine
Gating strategy DC - T cell co-culture, surface activation markers
F
Gated on Vβ11+ CD4+
Gating strategy DC - T cell co-culture, transcription factor staining
G
Gated on Vβ11+ CD4+
Gating strategy DC - T cell co-culture, intracellular cytokine staining
H
Gated on Vβ11+ CD4+

Supp Fig 1

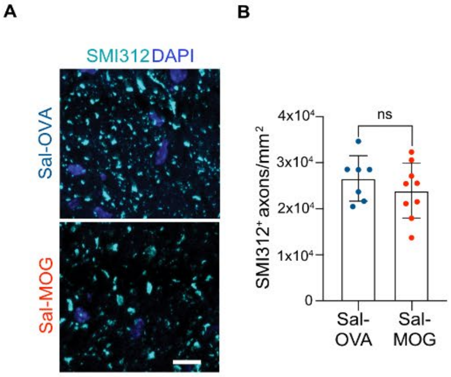

Supp Fig 2

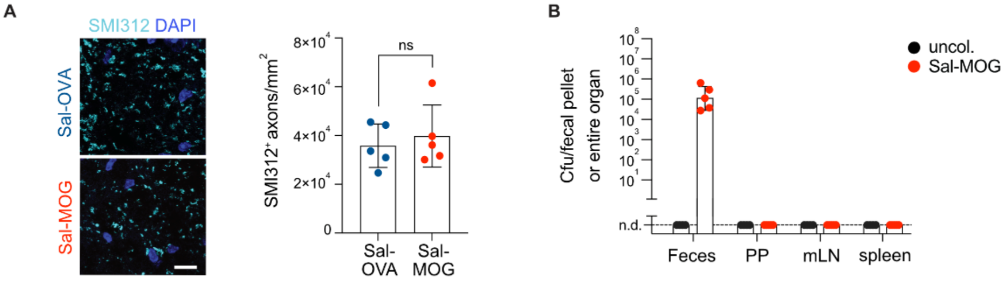

Supp Fig 3

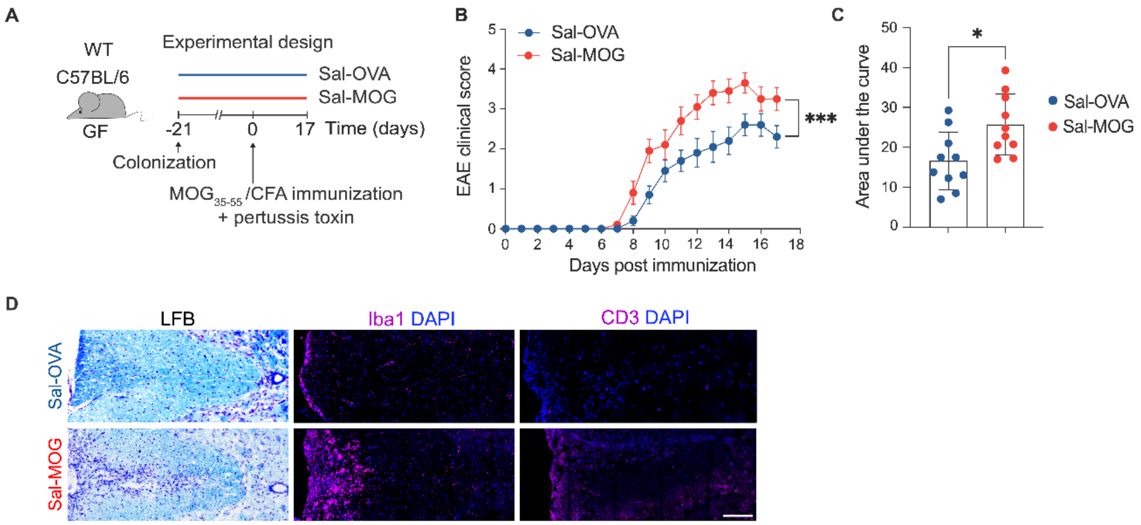
A
WT
C57BL/6
GF
Experimental design
Sal-OVA
Sal-MOG
-21
0
17
Time (days)
Colonization
MOG35-55/CFA immunization
+ pertussis toxin
B
EAE clinical score
Sal-OVA
Sal-MOG
***
Days post immunization
C
Area under the curve
*
Sal-OVA
Sal-MOG
D
LFB
Iba1 DAPI
CD3 DAPI
Sal-OVA
Sal-MOG

Supp Fig 4

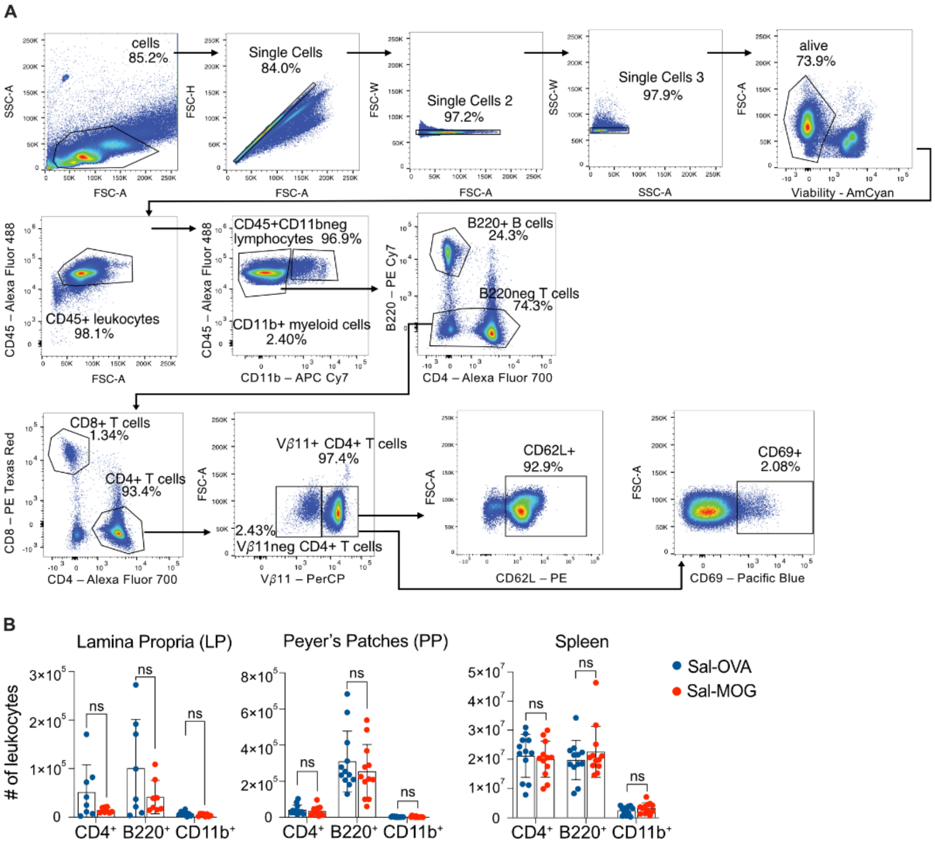
A
cells
85.2%
Single Cells
84.0%
Single Cells 2
97.2%
Single Cells 3
97.9%
alive
73.9%
SSC-A
FSC-A
FSC-H
FSC-W
SSC-W
Viability - AmCyan
CD45+ leukocytes
98.1%
CD45 – Alexa Fluor 488
CD45+CD11bneg
lymphocytes 96.9%
CD11b+ myeloid cells
2.40%
CD11b – APC Cy7
B220+ B cells
24.3%
B220neg T cells
74.3%
B220 – PE Cy7
CD4 – Alexa Fluor 700
CD8+ T cells
1.34%
CD4+ T cells
93.4%
CD8 – PE Texas Red
Vβ11+ CD4+ T cells
97.4%
2.43%
Vβ11neg CD4+ T cells
Vβ11 – PerCP
CD62L+
92.9%
CD62L – PE
CD69+
2.08%
CD69 – Pacific Blue
B
Lamina Propria (LP)
Peyer's Patches (PP)
Spleen
Sal-OVA
Sal-MOG
# of leukocytes
ns
CD4⁺
B220⁺
CD11b⁺

Supp Fig 5

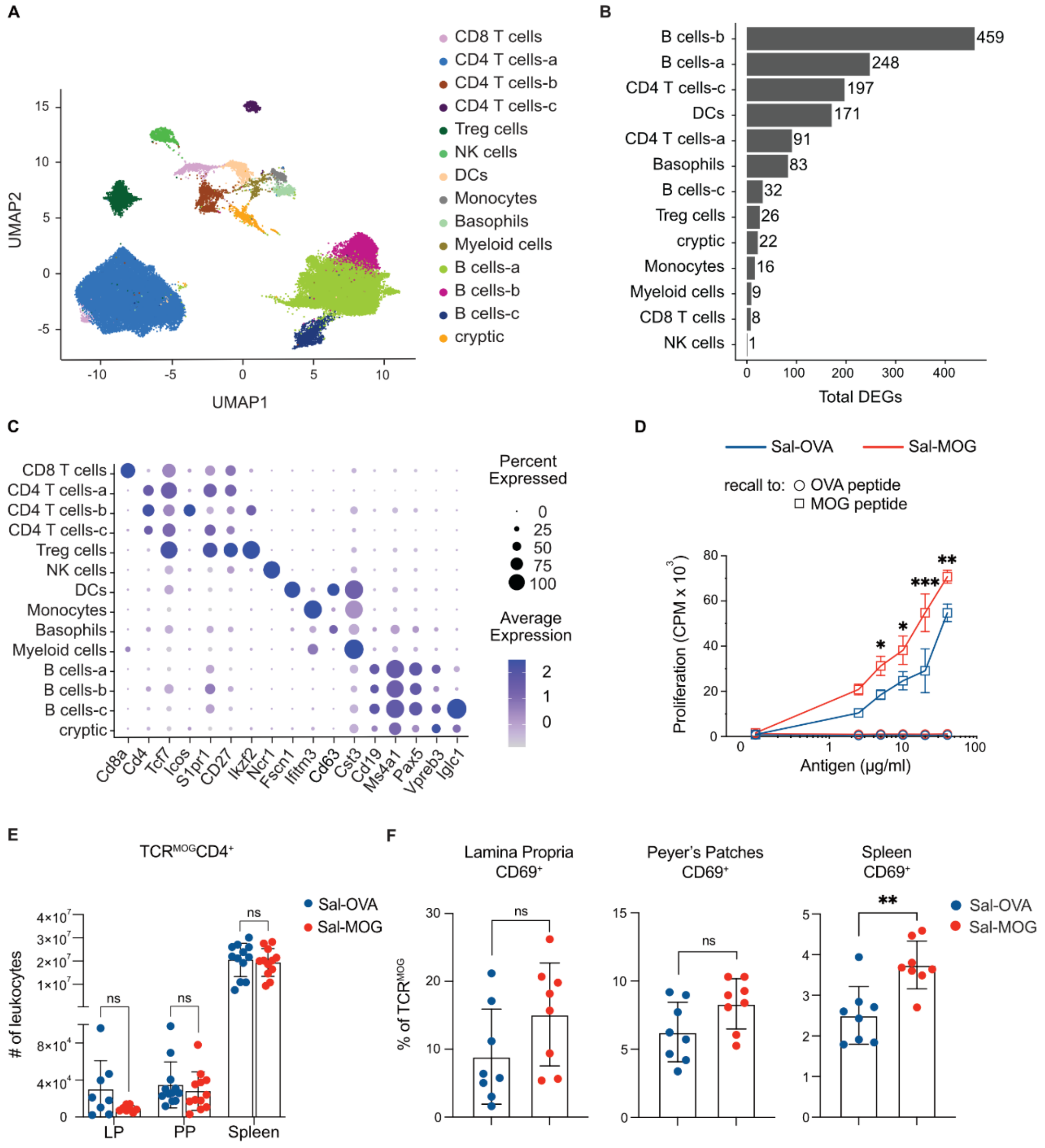
A
CD8 T cells
CD4 T cells-a
CD4 T cells-b
CD4 T cells-c
Treg cells
NK cells
DCs
Monocytes
Basophils
Myeloid cells
B cells-a
B cells-b
B cells-c
cryptic
UMAP2
UMAP1
B
Total DEGs
C
Percent Expressed
Average Expression
Cd8a Cd4 Tcf7 Icos S1pr1 CD27 Ikzf2 Ncr1 Fscn1 Ifitm3 Cd63 Cst3 Cd19 Ms4a1 Pax5 Vpreb3 Iglc1
D
Sal-OVA
Sal-MOG
recall to: OVA peptide
MOG peptide
Proliferation (CPM x 10^3)
Antigen (μg/ml)
E
TCR^MOG CD4+
# of leukocytes
LP
PP
Spleen
F
Lamina Propria CD69+
Peyer's Patches CD69+
Spleen CD69+
% of TCR^MOG
ns

Supp Fig 6
