# Supplementary figures and images for: Antigen-specific activation of gut immune cells drives autoimmune neuroinflammation
Source: Gut Microbes. 2025 Dec 24;18(1):2601430. doi: 10.1080/19490976.2025.2601430 (PMC12962552; doi:10.1080/19490976.2025.2601430)

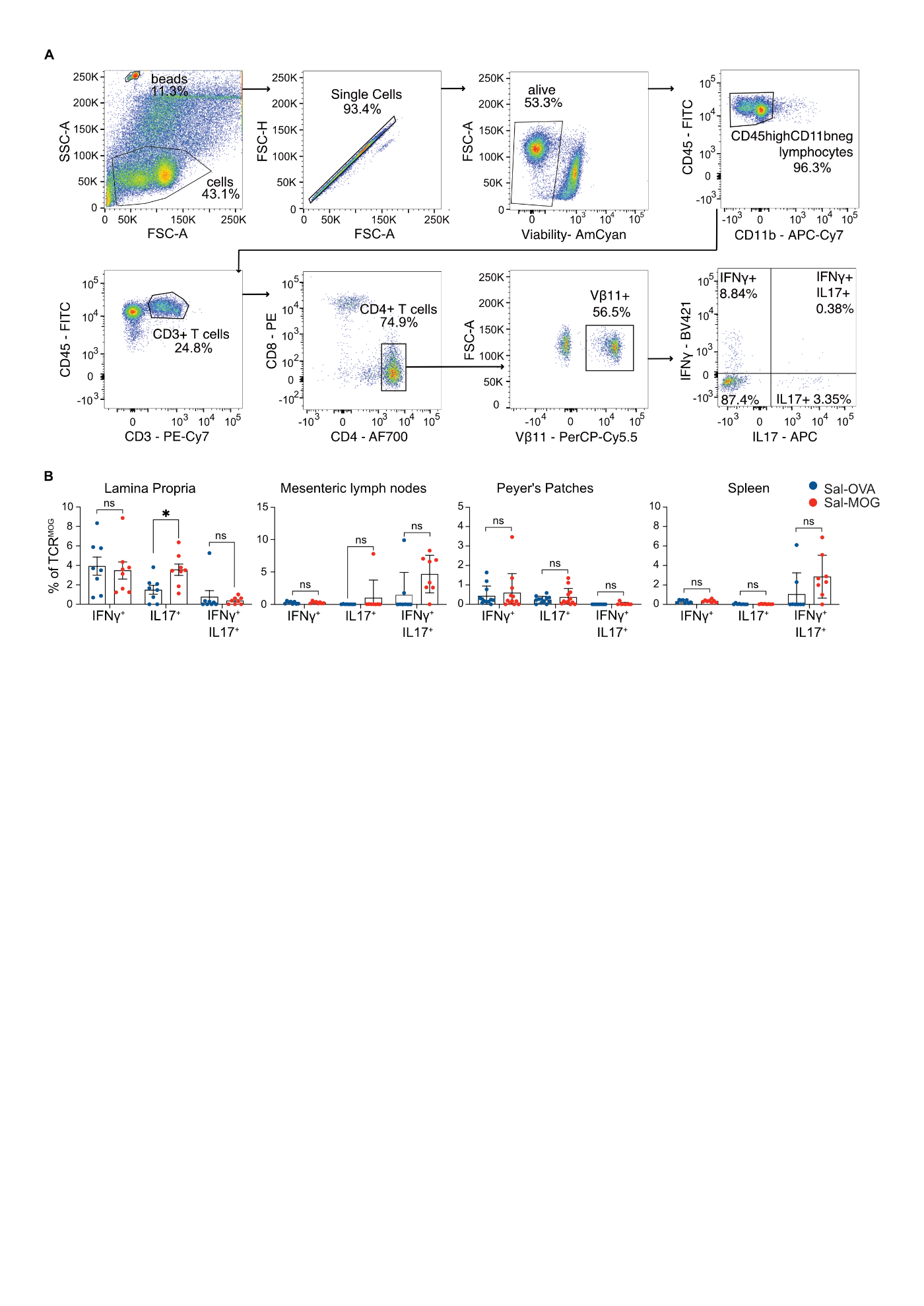


Supp Fig 7


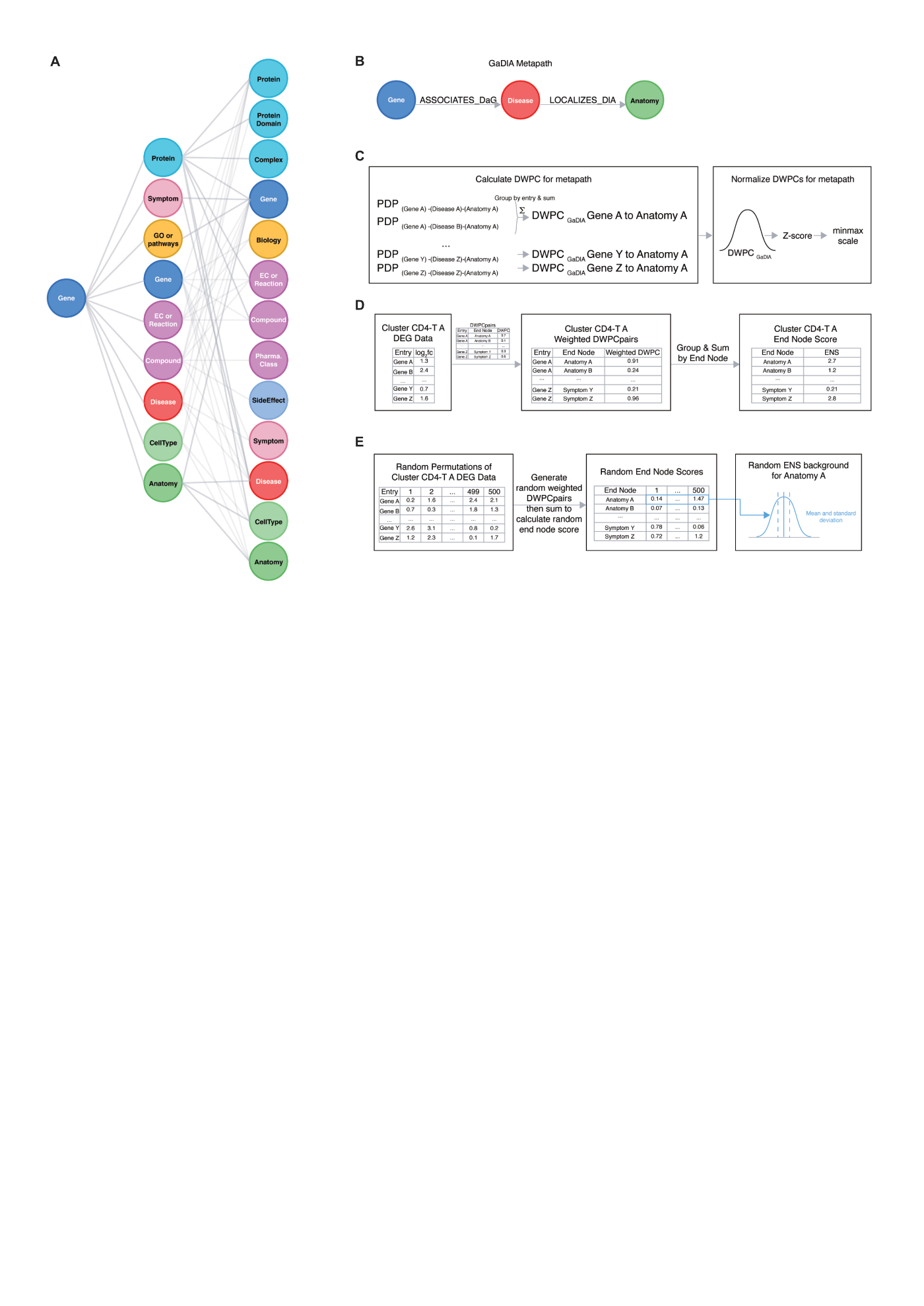


Supp Fig 8


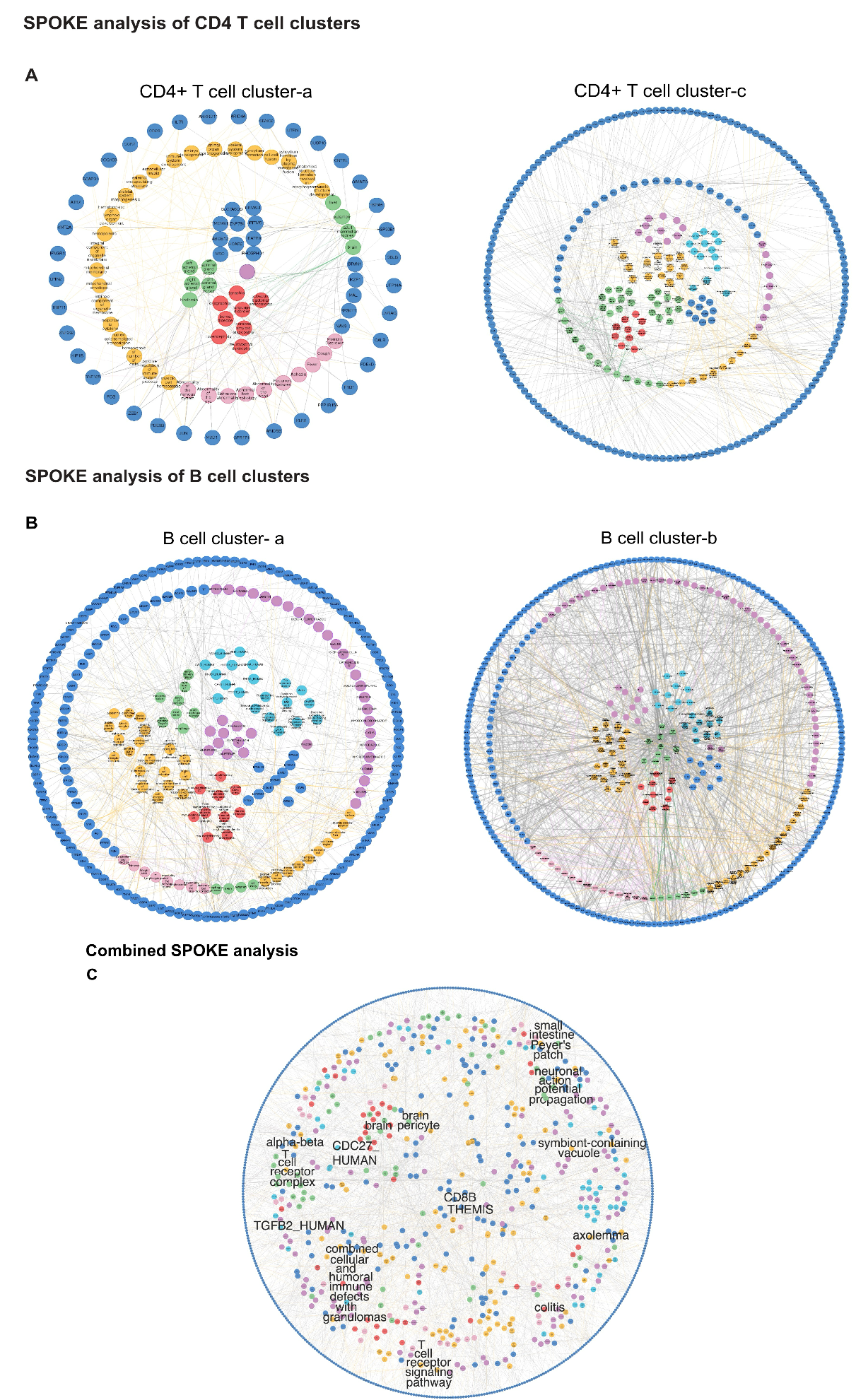


Supp Fig 9


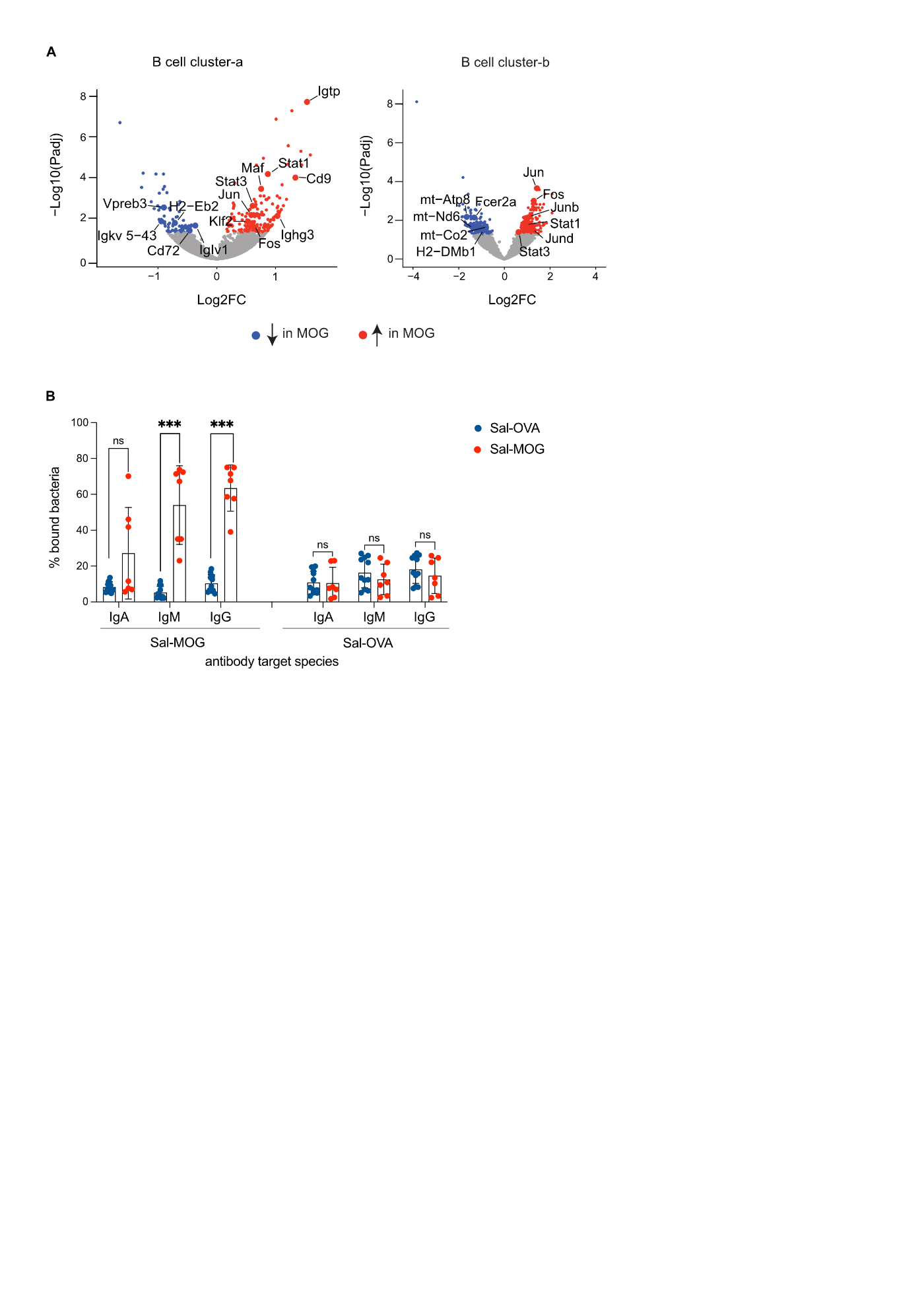


Supp Fig 10


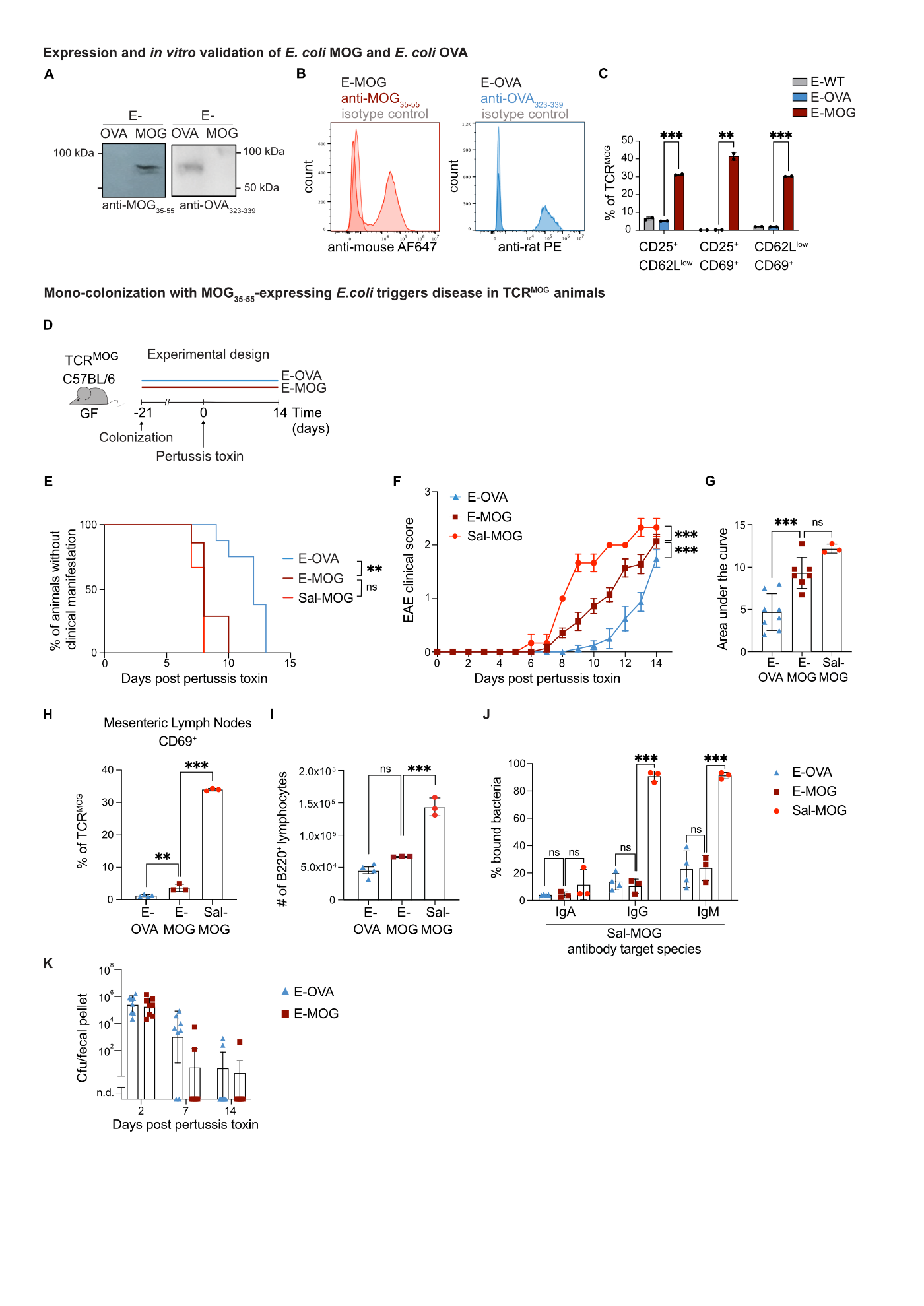


Supp Fig 11


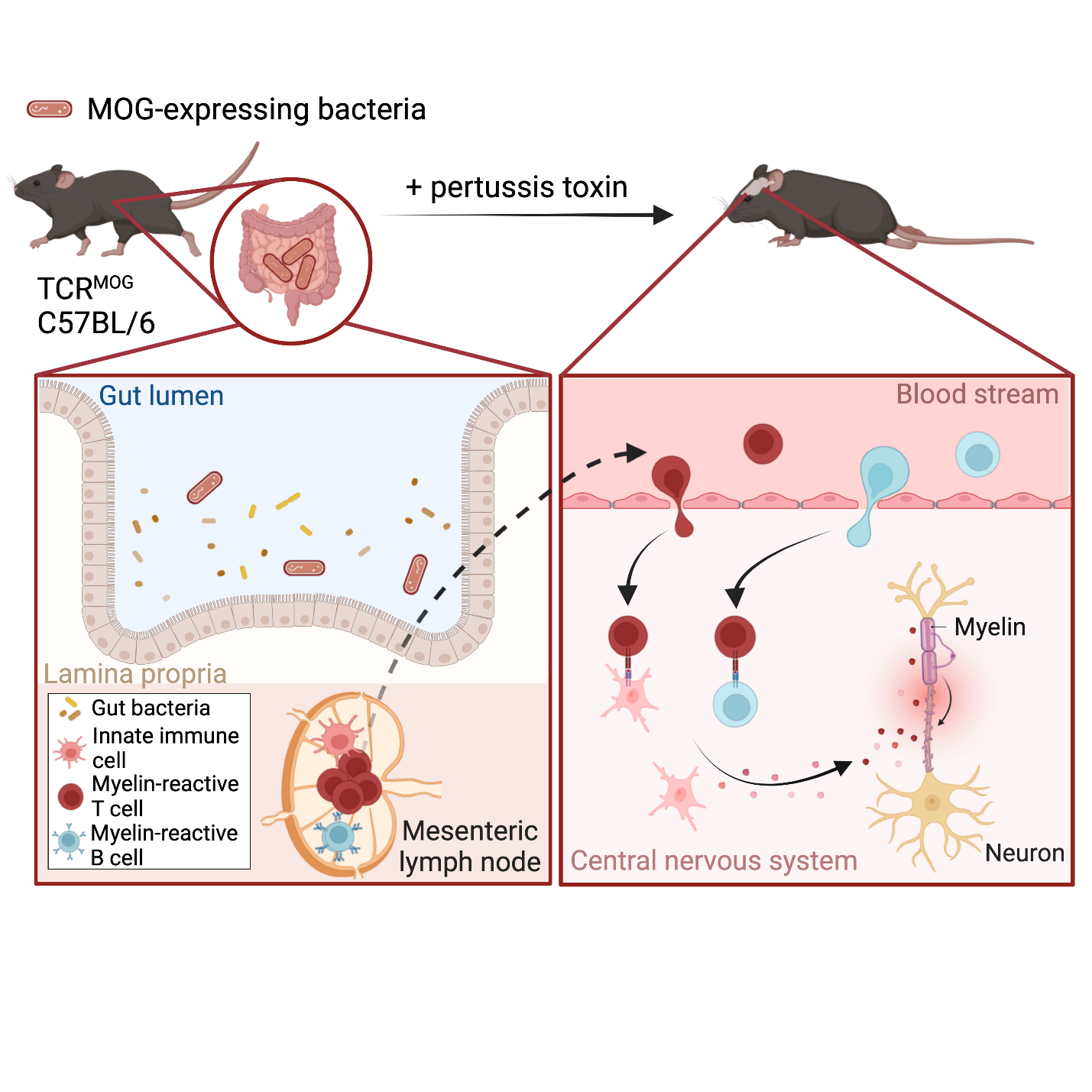


Supp Fig 12

Supplement: Supplementary Material — Supp_Fig_7-12.docx [file KGMI_A_2601430_SM2395.docx]
