# Supplementary material for: Antigen-specific activation of gut immune cells drives autoimmune neuroinflammation
Source: Gut Microbes. 2025 Dec 24;18(1):2601430. doi: 10.1080/19490976.2025.2601430 (PMC12962552; doi:10.1080/19490976.2025.2601430)
Supplement: Supplementary Material — Siewert_Berve_Supporting_Information.docx [file KGMI_A_2601430_SM2393.docx]

**Supplementary Information for**

**Antigen-specific activation of gut immune cells drives autoimmune neuroinflammation**

Lena K. Siewert^1,2,3,^†, Kristina Berve^1,2,^†, Elisabeth Pössnecker^1,2^, Julia Dyckow^4,18^, Amel Zulji^4^, Ryan Baumann^5^, Aida Munoz-Blazquez^1,2^, Gurumoorthy Krishnamoorthy^6,7^, David Schreiner^1,2^, Sharon Sagan^5,15^, Charlotte Nelson^5^, Joseph J. Sabatino, Jr.^5^, Kazuki Nagashima^8,9,10,11^, Médéric Diard^12^, Andrew J. Macpherson^13,14^, Stephanie C. Ganal-Vonarburg^13,14^, Michael A. Fischbach^8,9,10,11^, Scott S. Zamvil^5,15^, Lucas Schirmer^4,16,17,18^, Sergio E. Baranzini^5,19,20,^†, Anne-Katrin Pröbstel^1,2,6,7,^†,*

^1^Departments of Neurology, Biomedicine and Clinical Research, University Hospital and University of Basel, Basel, Switzerland
^2^Research Center for Clinical Neuroimmunology and Neuroscience Basel (RC2NB), University Hospital and University of Basel, Basel, Switzerland
^3^European Society for Clinical Microbiology and Infectious Diseases (ESCMID), Basel, Switzerland
^4^Department of Neurology, Medical Faculty Mannheim, Heidelberg University, Mannheim, Germany
^5^Department of Neurology & Weill Institute for Neurosciences, University of California, San Francisco, US
^6^Center of Neurology, Department of Neuroimmunology, University Hospital and University Bonn, Bonn, Germany
^7^German Center for Neurodegenerative Diseases (DZNE), Bonn, Germany
^8^Department of Bioengineering, Stanford University, Stanford, CA 94305, US
^9^Department of Microbiology and Immunology, Stanford University School of Medicine, Stanford University, Stanford, CA 94305, US
^10^ChEM-H Institute, Stanford University, Stanford, CA 94305, US
^11^Chan Zuckerberg Biohub, San Francisco, CA 94158, US
^12^Biozentrum, University of Basel, Basel, Switzerland
^13^Department of Visceral Surgery and Medicine, Inselspital, Bern University Hospital, University of Bern, Bern, Switzerland
^14^Department for BioMedical Research, University of Bern, Bern, Switzerland
^15^Program in Immunology, University of California, San Francisco, US
^16^Mannheim Center for Translational Neuroscience, Medical Faculty Mannheim, Heidelberg University, Mannheim, Germany
^17^Mannheim Institute for Innate Immunoscience, Medical Faculty Mannheim, Heidelberg University, Mannheim, Germany
^18^Interdisciplinary Center for Neurosciences, Heidelberg University, Heidelberg, Germany
^19^Institute for Human Genetics, University of California, San Francisco, US
^20^Graduate Program in Bioinformatics, University of California, San Francisco, US

† These authors contributed equally to this work

* Corresponding author: anne-katrin.proebstel@ukbonn.de

**This PDF file includes:**

Supplementary Figures and -Legends 1 to 12

**Supplementary Figures**

**Supplementary Fig. 1. *In vitro* validation of engineered bacterial strains**. (A, B) Expression validation of MOG_35-55_ and OVA_323-339_ fusion peptide constructs of *Salmonella* by Western Blot (A) and flow cytometry of live bacteria (B). (C-E) Co-cultivation assay of respective T cells and *in vitro* primed CD45.1 DCs (stimulation with MOG or OVA peptide or heat-inactivated *Salmonella* strains). (C) Surface marker analysis of OVA-reactive OT-II T cells. (D, E) Quantification of the flow cytometry analysis of surface marker (D), transcription factor (E, left) and cytokine (E, right) levels in TCR^MOG^ T cells after co-culture. (F-H) Flow cytometry gating strategy for the *in vitro* analysis of respective transgenic TCR^MOG^ T cells co-cultured with peptide or heat-inactivated bacteria primed CD45.1 DCs. (F) Surface activation marker staining presented in (Fig. 1C, D); (G) intracellular transcription factor staining presented in (E, left); (H) intracellular cytokine staining presented in (E, right). Experiments in (A-H) show examples of at least two independent experiments. (C-E) Unpaired *t*-test, Holm-Šídák method. Mean ± SD. ns = not significant; * p < 0.05; ** p < 0.01; *** p < 0.001. Sal = *Salmonella,* MOG = myelin oligodendrocyte glycoprotein, OVA = ovalbumin. Related to Fig. 1.

**Supplementary Fig. 2. Assessment of axonal damage in colonized SPF TCR^MOG^ mice**. (A) Representative histopathological analysis of SMI312^+^ axons in dorsal fiber tracts of the spinal cord for experiments presented in (Fig. 1G-L). Scale bar: 10 µm. (B) Quantification of SMI312^+^ axons from (A). Pooled analysis of two independent experiments with at least n = 3 mice per group. Unpaired *t*-test. Mean ± SD. Each dot represents one mouse. ns = not significant. Sal = *Salmonella,* MOG = myelin oligodendrocyte glycoprotein, OVA = ovalbumin. Related to Fig. 1.

**Supplementary Fig. 3. Histopathological analysis of axonal damage in mono-colonized germ-free (GF) TCR^MOG^ mice and gut-specific colonization controls.** (A) Histopathological analysis of the dorsal fiber tracts in the spinal cord of mono-colonized TCR^MOG^ mice (left). Scale bar: 10 µm. Quantification of SMI312^+^ axons (right). N=5 mice. Mann-Whitney test. (B) Post-mortem colonization controls for mono-colonized mice from (Fig. 2G-J). Colony forming units (cfu) were assessed by limited dilution plating for feces, Peyer’s patches (PP), mesenteric lymph node (mLN) and spleen at 14 days post pertussis toxin treatment. N= 5 mice. (A, B) Mean ± SD. Each dot represents one mouse. ns = not significant. uncol. = uncolonized. Sal = *Salmonella,* MOG = myelin oligodendrocyte glycoprotein, OVA = ovalbumin. Related to Fig. 2.

**Supplementary Fig. 4. Mono-colonization with MOG_35-55_-expressing *Salmonella* exacerbates EAE in GF wildtype mice.** (A) Experimental set-up of the experiment shown in (B-D). GF wildtype (WT) C57BL/6 mice were mono-colonized with Sal-OVA or -MOG 21 days prior to immunization with MOG_35-55_/CFA emulsion and pertussis toxin injection. (B) EAE scores of Sal-MOG versus -OVA mono-colonized WT mice. (C) Overall disease severity as determined by area under the curve analysis between Sal-MOG and Sal-OVA mono-colonized WT mice with EAE. (D) Representative histopathological analysis in the spinal cord (dorsal fiber tracts) of mono-colonized WT EAE mice showing luxol fast blue (LFB)-stained myelin (left), Iba1^+^ microglia/macrophages (center) and CD3^+^ T cells (right). Scale bar: 100 µm. Experiments in (B, C) show pooled analysis from two independent experiments with n = 5 mice per group/experiment. (B) Repeated-measures two-way ANOVA; mean ± SEM. (C) Unpaired *t*-test; mean ± SD. Each dot represents one mouse. * p < 0.05; *** p < 0.001; ns = not significant. Sal = *Salmonella,* MOG = myelin oligodendrocyte glycoprotein, OVA = ovalbumin, EAE = experimental autoimmune encephalitis.

**Supplementary Fig. 5. Immune cell phenotyping of GF TCR^MOG^ mice mono-colonized with MOG_35-55_-expressing *Salmonella.*** Flow cytometry gating strategy for the analysis of different immune cell subsets in Peyer’s patches, lamina propria, mesenteric lymph node and spleen of mono-colonized TCR^MOG^ mice as displayed in (B) and (Fig. 3A, E, F). (B) Quantification of CD4^+^, B220^+^ and CD11b^+^ cells in the respective immune compartments. Experiments in (B) show pooled analysis of two (lamina propria) or three (PP, spleen) independent experiments with at least n = 4 mice per group/experiment. Unpaired *t*-test. Mean ± SD. Each dot represents one mouse. ns = not significant. Sal = *Salmonella,* MOG = myelin oligodendrocyte glycoprotein, OVA = ovalbumin. Related to Fig. 3.

**Supplementary Fig. 6. Single-cell RNA sequencing of mono-colonized mice and T cell activation phenotype across immune compartments.**

(A) Uniform manifold approximation and projection (UMAP) visualization representing 58698 single cell RNA sequencing profiles from the mesenteric lymph nodes of Sal-MOG and OVA mono-colonized TCR^MOG^ mice. (B) Total number of differentially expressed genes per cluster shown in A. (C) Expression and specificity of genes used for cell type annotation in (A). (D) *Ex vivo* proliferation analysis of splenocytes from mono-colonized TCR^MOG^ mice 5 weeks post-colonization and 2 weeks post pertussis toxin treatment. [3H]-thymidine incorporation (CPM) was measured upon stimulation with either MOG_35-55_- or OVA_323-339_-peptide. (E) Quantitative flow cytometry analysis of TCR^MOG^ CD4^+^ T cells in lamina propria (LP), Peyer’s patches and spleen of mono-colonized Sal-MOG and Sal-OVA mice. (F) Flow cytometry analysis of CD69 surface expression in TCR^MOG^ CD4^+^ T cells isolated from lamina propria (left), Peyer’s patches (center) and spleen (right) from Sal-MOG vs. Sal-OVA mono-colonized mice. (D) shows one experiment with n = 4 mice/group. Experiments in (E) show pooled analysis of two (LP) and three (PP, spleen) independent experiments with n = 4 mice per group/experiment. Experiments in (F) show pooled analysis of two independent experiments with n = 4 mice per group/experiment. (D) Ordinary two-way ANOVA followed by Tukey’s multiple comparisons test (significant differences are depicted for MOG peptide only); mean ± SEM. (E, F) Unpaired *t*-test; mean ± SD. Each dot represents one mouse. ns = not significant. * p < 0.05; ** p < 0.01; *** p < 0.001. Sal = *Salmonella,* MOG = myelin oligodendrocyte glycoprotein, OVA = ovalbumin, DEG = differentially expressed genes. Related to Fig. 3 and Tables Z1 – 4, Zenodo.

**Supplementary Fig. 7. Cytokine profile in different peripheral immune compartments of mono-colonized GF TCR^MOG^ mice.** (A) Flow cytometry gating strategy for the analysis of cytokine expression within TCR^MOG^ CD4^+^ T cells of mono-colonized TCR^MOG^ mice as displayed in (B). (B) Quantification of the baseline (3 weeks post colonization) cytokine expression levels of IFN-γ, IL17^+^ or IFN-γ/IL17^+^ TCR^MOG^ T cells in lamina propria (far left), mesenteric lymph node (center left), Peyer’s patches (center right) and spleen (far right). Experiments in (B) show pooled analysis of two independent experiments with n = 4 mice per group/experiment. Unpaired *t*-test, Holm-Šídák method; mean ± SEM. Each dot represents one mouse. ns = not significant. * p < 0.05. Sal = *Salmonella,* MOG = myelin oligodendrocyte glycoprotein; OVA = ovalbumin.

**Supplementary Fig. 8. SPOKE knowledge graph and analysis to interpret single-cell RNA sequencing signatures**. SPOKE is a massive knowledge graph that includes more than 40 databases of interest in biology and medicine. (A, B) Computed degree-weighted path counts (DWPC) (A) to score the connections between genes that were significantly upregulated in each cluster to any SPOKE end node within a path length of three (B). (C) Calculation of DWPCs for a given metapath. (D) The DWPCs were grouped by entry-end node pairs and summed to calculate DWPC_pair_. (E) The weighted DWPC_pair_ was grouped by end node and summed up to score the importance of each end node for the given cluster. For visualization, we extracted the nodes with the highest values for each analyzed cluster and represented them in a connected sub-graph where the entry nodes (differentially expressed genes) are depicted as a circle and the highest scoring nodes of different types are displayed inside. ENS = End node score; DWPC = degree-weighted path counts. Related to Supplementary Fig. 9.

**Supplementary Fig. 9. SPOKE analysis of T and B cell clusters within the mesenteric lymph node.** SPOKE analysis of (A) CD4 T cell cluster-a (left) and CD4 T cell cluster-c (right) highlighting biological processes from gene ontology (orange), neurological diseases (red), anatomical regions (green) and symptoms (pink). (B) SPOKE analysis of B cell cluster-a (left) highlighting concepts like T cell receptor signaling (orange), immunodeficiency (red), and Peyer’s patches (green) and B cell cluster-b (right) highlighting concepts like axon regeneration (orange), segments of the spinal cord (green), and colon inflammation (red). (C) Combined SPOKE analysis of CD4 T cell clusters-a and -c and B cell clusters-a and -b. Experiment was performed using n = 3 mice per group. Related to Supplementary Fig. 8 and Table Z4, Zenodo. The network shown in this figure is available for download on Zenodo (<https://doi.org/10.5281/zenodo.16927590>) and can be explored interactively using Cytoscape (<https://cytoscape.org/>).

**Supplementary Fig. 10. B cell activation and antibody production in mono-colonized TCR^MOG^ mice.**

(A) Volcano plots showing differentially expressed genes between Sal-MOG and -OVA mono-colonized mice within B cell cluster-a (left) and cluster-b (right). Red and blue dots represent dysregulated genes according to a cutoff of Padj = 0.05. Experiment was performed using n = 3 mice per group. (B) Bacterial flow cytometry analysis of serum antibodies in Sal-MOG and -OVA mono-colonized mice, 5 weeks post colonization and 2 weeks post pertussis treatment. Shown is the pooled analysis of two independent experiments with at least n = 3 mice per group/experiment. Unpaired *t*-test, Holm-Šídák method; mean ± SD. Each dot represents one mouse. ns = not significant; *** p < 0.001. Sal = *Salmonella,* MOG = myelin oligodendrocyte glycoprotein, OVA = ovalbumin; Padj = adjusted P value, FC = fold change.

**Supplementary Fig. 11. Validation of *E. coli* construct.** (A) Expression validation of MOG_35-55_ and OVA_323-339_ fusion peptide constructs of *E. coli* by Western Blot (A) and flow cytometry of live bacteria (B). (C) Co-cultivation assay of TCR^MOG^ cells and *in vitro* primed CD45.1 DCs (stimulation with MOG or OVA peptide or heat-inactivated *E. coli* strains). The resulting T cell activation was analyzed by flow cytometry. (D) Experimental set-up of the experiments shown in (E-G). Mono-colonization of GF TCR^MOG^ mice with E-OVA or -MOG (in comparison to Sal-MOG) and additional pertussis toxin administration 21 days post colonization. (E) Percentage of mice without clinical manifestation during the observed time period. (F) EAE clinical scores of mono-colonized TCR^MOG^ mice. (G) Overall disease severity as determined by area under the curve analysis between E-MOG, E-OVA and Sal-MOG. (H) Flow cytometry analysis of CD69 surface expression on TCR^MOG^ T cells isolated from mesenteric lymph nodes from diseased Sal-MOG, E-MOG and E-OVA colonized mice harvested 2 weeks post pertussis toxin treatment. (I) Quantitative flow cytometry analysis of B220^+^ B cells in mesenteric lymph nodes isolated from diseased Sal-MOG, E-MOG and E-OVA mono-colonized mice harvested 2 weeks post pertussis toxin treatment. (J) Bacterial flow cytometry analysis of serum antibodies of E-MOG and -OVA colonized mice, 2 weeks post pertussis treatment. Sal-MOG served as a surrogate to detect anti-MOG_35-55_ antibodies. (K) Counted colony forming units (cfu) from an entire fecal pellet at indicated time points after colonization from mice shown in (Fig. 4D-F). Experiment in (C) shows example of two independent experiments. Experiments in (E-G) show pooled analysis of two independent experiments with n = 4 mice per group/experiment; except from Sal-MOG: n = 3 mice. Experiments in (H-J) show example of at least two independent experiments with at least n = 3 mice/group. Experiment in (K) shows pooled analysis of two independent experiments with n = 4 mice per group/experiment. (C) Multiple unpaired *t*-test, Holm-Šídák method. (E) Log-rank test. (F) Repeated-measures two-way ANOVA. (G) Ordinary one-way ANOVA followed by Tukey’s multiple comparisons test. (H-I) Ordinary one-way ANOVA followed by Tukey’s multiple comparisons test. (J) Ordinary two-way ANOVA followed by Tukey’s multiple comparisons test. (C, G-K) Mean ± SD. (F) Mean ± SEM. (G-K) Each triangle/square/dot represents one mouse. ns = not significant; * p < 0.05; ** p < 0.01; *** p < 0.001. Sal = *Salmonella,* MOG = myelin oligodendrocyte glycoprotein, OVA = ovalbumin, EAE = experimental autoimmune encephalitis. Related to Fig. 4.

**Supplementary Fig. 12:** **Proposed model of antigen-specific activation of encephalitogenic immune cells in the gut associated lymphoid tissues leading to neuroinflammation.** Gut colonization with myelin oligodendrocyte glycoprotein (MOG)-expressing bacteria leads to antigen-specific activation of autoreactive immune cells in the mesenteric lymph nodes in a T-cell receptor transgenic murine model (TCR^MOG^). Opening of the blood-brain barrier with pertussis toxin results in the infiltration of MOG-reactive immune cells into the central nervous system (CNS), exacerbating CNS inflammation and clinical disease.
